# Supplementary material for: Loss of cohesin regulator PDS5A reveals repressive role of Polycomb loops
Source: Nat Commun. 2023 Dec 9;14:8160. doi: 10.1038/s41467-023-43869-w (PMC10710464; doi:10.1038/s41467-023-43869-w)

Loss of Cohesin regulator PDS5A reveals repressive role of Polycomb loops.

Daniel Bsteh[1,2,3,4], Hagar F. Moussa[1,2,5], Georg Michlits[1,2,6], Ramesh Yelagandula[1,7], Jingkui Wang[1,8], Ulrich Elling[1], Oliver Bell[1,3*]

Supplementary Information

Supplementary Figures 1-6
Supplementary Tables 1-3

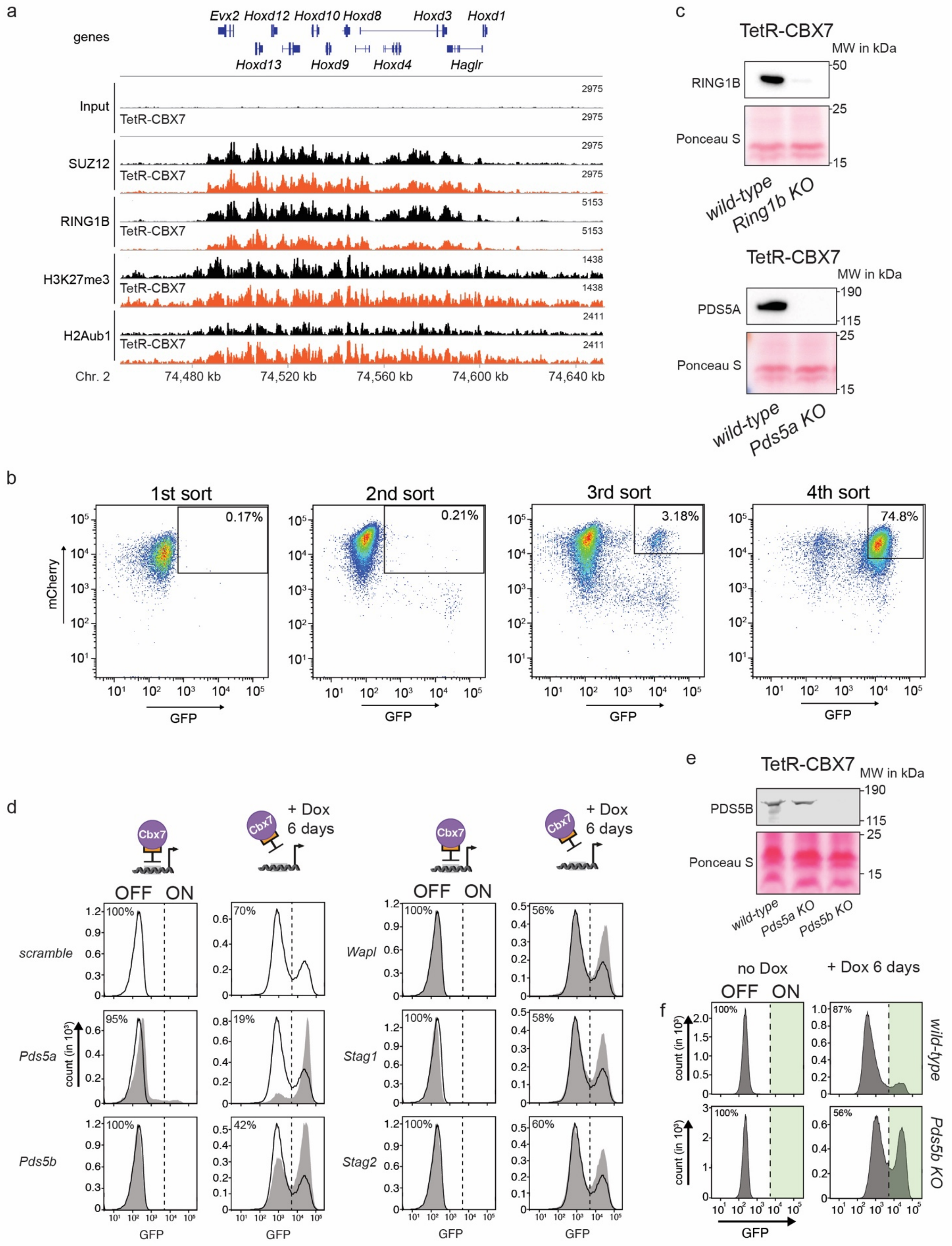
a
genes
Evx2 Hoxd12 Hoxd10 Hoxd8 Hoxd3 Hoxd1
Hoxd13 Hoxd9 Hoxd4 Haglr
Input
SUZ12
RING1B
H3K27me3
H2Aub1
TetR-CBX7
Chr. 2
74,480 kb 74,520 kb 74,560 kb 74,600 kb 74,640 kb
c
TetR-CBX7
MW in kDa
RING1B
Ponceau S
wild-type
Ring1b KO
PDS5A
Pds5a KO
b
1st sort 2nd sort 3rd sort 4th sort
0.17% 0.21% 3.18% 74.8%
mCherry
GFP
e
PDS5B
Pds5b KO
d
Cbx7
+ Dox 6 days
OFF ON
scramble
Pds5a
Pds5b
Wapl
Stag1
Stag2
count (in 10³)
f
no Dox
+ Dox 6 days
wild-type
Pds5b KO

Supplementary Fig. 1. CRISPR screen of cPRC1-dependent gene silencing reveals *Pds5a*. a, ChIP-CapSeq was used to compare levels of PcG proteins and histone modifications before (black) and after TetR-Cbx7 expression (orange). Capture oligos (Baits) were designed against the reporter locus and 24 reference loci including Polycomb and non-Polycomb target genes (Supplementary Table 3). Shown is a genomic screenshot of the *Hoxd* gene cluster. **b,** Flow cytometry scatter plots of CRISPR screen sorting scheme to enrich for GFP-positive reporter ESCs. X-axis shows reporter GFP expression and y-axis shows expression of mCherry-tagged TetR-Cbx7. Shown are gates and fraction of GFP-positive cells (in %) in each sort. **c,** Western blots compare RING1B and PDS5A expression in parental TetR-Cbx7 and clonal CRISPR mutant reporter ESCs. **d,** Flow cytometry histograms of GFP signal before (left) and after 6 days of Dox-dependent reversal of TetR-CBX7 tethering (right) in Polycomb reporter ESCs transduced with Cas9/sgRNAs targeting genes encoding different cohesin release factors (gray). Polycomb reporter ESCs transduced with Cas9 and scramble sgRNA serves as control (black line). Percentages indicates fraction of GFP-negative Polycomb reporter ESCs.

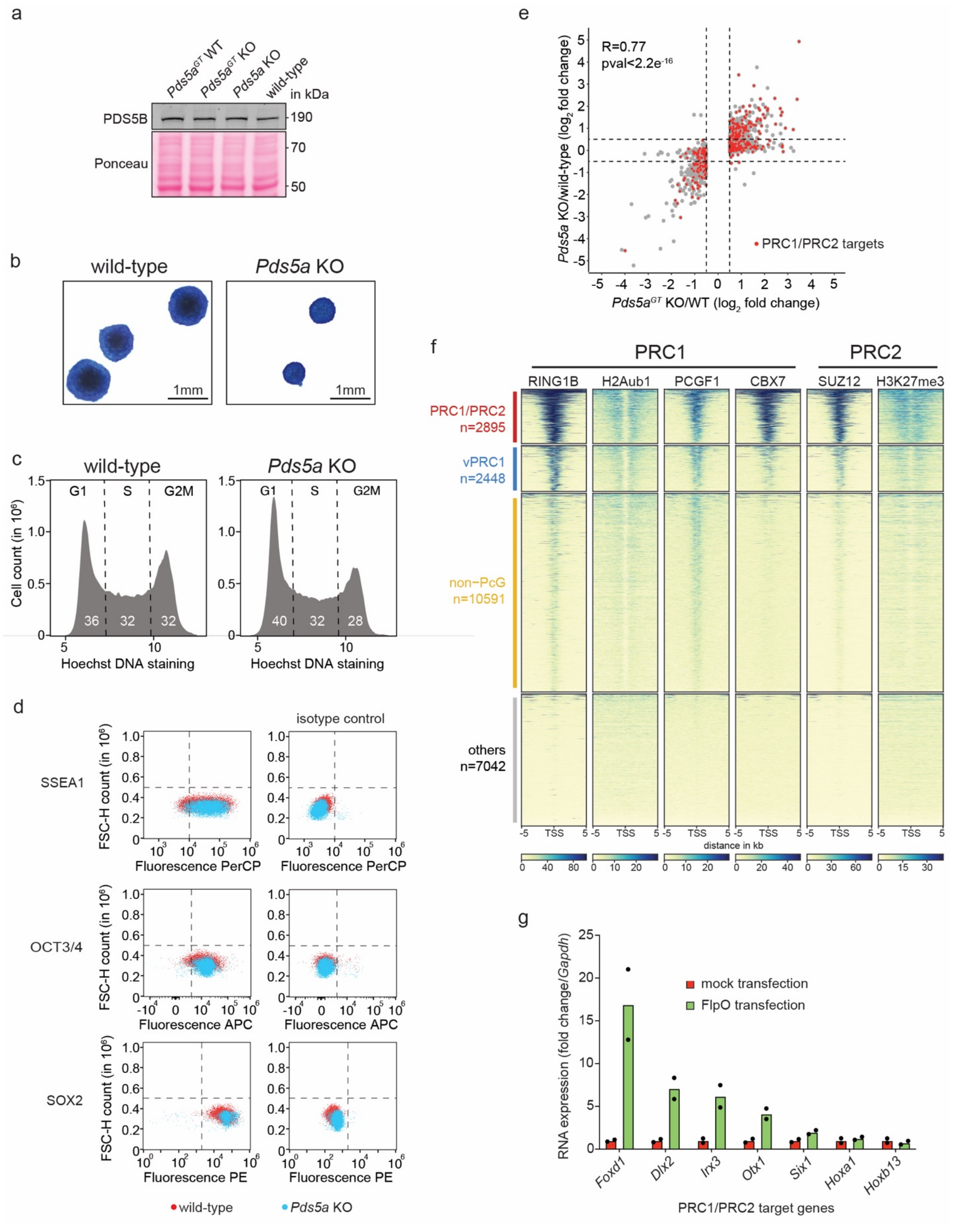

a
Pds5a^GT WT
Pds5a^GT KO
Pds5a KO
wild-type
in kDa
PDS5B
190
Ponceau
70
50
b
wild-type
Pds5a KO
1mm
1mm
c
wild-type
Pds5a KO
G1
S
G2M
36
32
32
40
32
28
Cell count (in 10^6)
Hoechst DNA staining
d
isotype control
SSEA1
OCT3/4
SOX2
FSC-H count (in 10^6)
Fluorescence PerCP
Fluorescence APC
Fluorescence PE
wild-type
Pds5a KO
e
R=0.77
pval<2.2e^-16
Pds5a KO/wild-type (log2 fold change)
Pds5a^GT KO/WT (log2 fold change)
PRC1/PRC2 targets
f
PRC1
PRC2
RING1B
H2Aub1
PCGF1
CBX7
SUZ12
H3K27me3
PRC1/PRC2
n=2895
vPRC1
n=2448
non−PcG
n=10591
others
n=7042
TSS
distance in kb
g
RNA expression (fold change/Gapdh)
mock transfection
FlpO transfection
Foxd1
Dlx2
Irx3
Otx1
Six1
Hoxa1
Hoxb13
PRC1/PRC2 target genes

Supplementary Fig. 2. PDS5A deletion does not impair ESC self-renewal and proliferation. a, Western blot of PDS5B expression in *Pds5a*^{GT} KO and *Pds5a*^{GT} WT ESCs and in *Pds5a* KO and wild-type ESCs. **b,** Alkaline phosphatase staining of wild-type and *Pds5a* KO ESCs. **c,** Flow cytometry histogram shows cell cycle distribution of wild-type and *Pds5a* KO ESCs based on Hoechst DNA staining. Numbers indicate fraction of ESC population in G1, S and G2M phase (in %). **d,** Flow cytometry scatter plots compares of stem cell marker expression in wild-type (red) and *Pds5a* KO (blue) ESCs. Right panel shows corresponding antibody isotype controls. **e,** Pairwise correlation plot of $\log_2$ fold changes in expression of DEGs (1029) in *Pds5a*^{GT} KO ESCs and *Pds5a* KO ESCs. R is Pearson correlation coefficient. PRC1/PRC2 target genes are indicated in red. **f,** cChIP-seq heatmaps of RING1B, H2Aub1, SUZ12 and H3K27me3 in wild-type ESCs. Enrichment signal is plotted around the TSS (+/- 5 kb) and clustered based on gene class annotation: PRC1/PRC2 target genes (red; n=2895), vPRC1 target genes (blue; n=2448), non-PcG genes (yellow; n=10591) and others (grey; n= 7042). **g,** RT-qPCR expression analysis of selected PRC1/PRC2 target genes in *Pds5a*^{GT} WT ESCs 72 hours after transfection with vector expressing FlpO recombinase inducing gene trap inversion or empty vector (mock). Shown are fold-changes normalized by *Gapdh* of two independent transfection experiments. Source data are provided as a Source Data file.

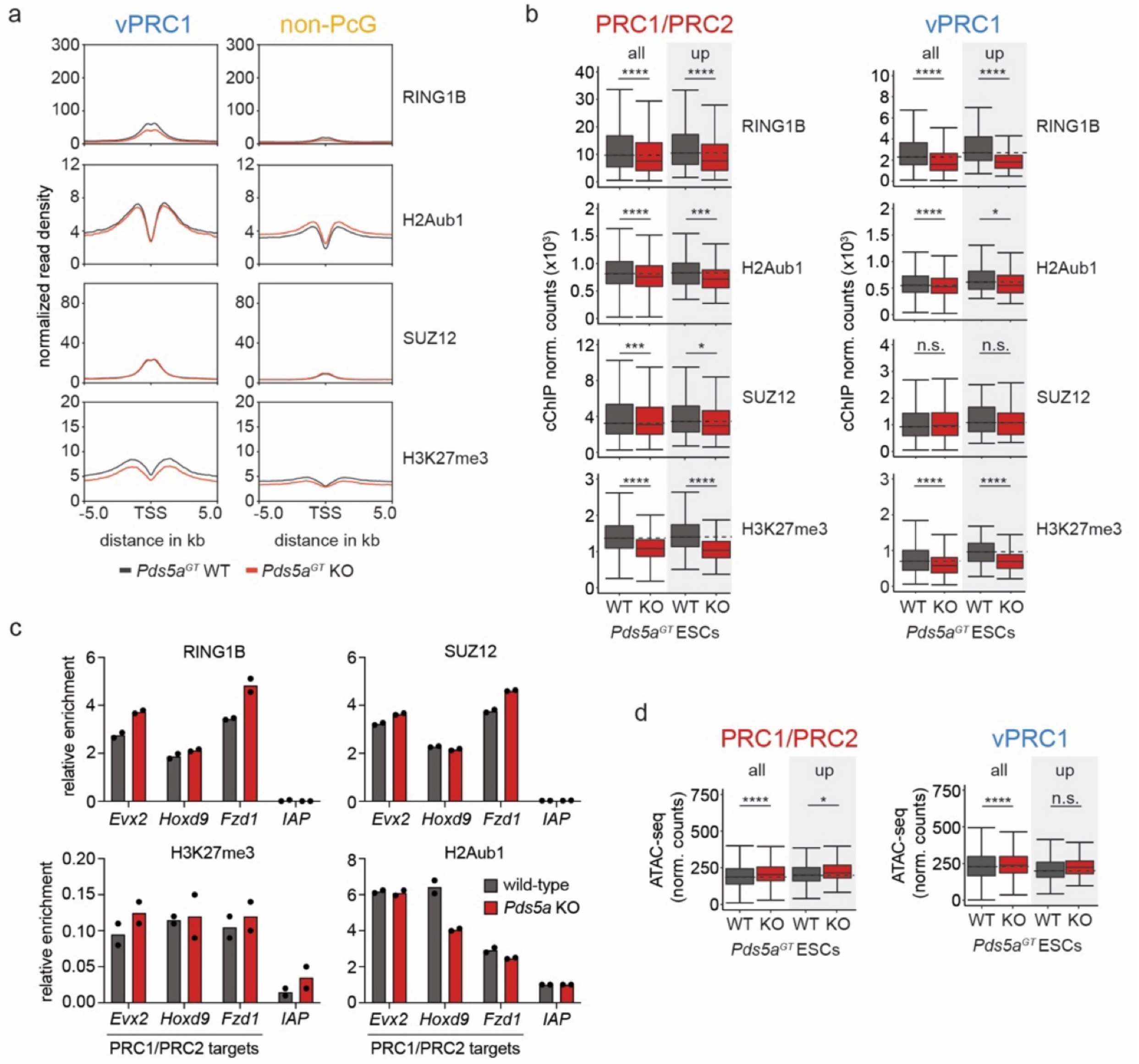
a
vPRC1
non-PcG
RING1B
H2Aub1
SUZ12
H3K27me3
normalized read density
distance in kb
Pds5a^GT WT
Pds5a^GT KO
b
PRC1/PRC2
vPRC1
all
up
cChIP norm. counts (x10³)
WT KO WT KO
Pds5a^GT ESCs
c
RING1B
SUZ12
H3K27me3
H2Aub1
relative enrichment
Evx2 Hoxd9 Fzd1 IAP
PRC1/PRC2 targets
wild-type
Pds5a KO
d
PRC1/PRC2
vPRC1
ATAC-seq (norm. counts)
n.s.

Supplementary Fig. 3. PDS5A deletion has minimal effect on Polycomb chromatin domains. a, Meta plots show average RING1B, H2Aub1, SUZ12 and H3K27me3 cChIP-seq signals at vPRC1 and non-PcG target genes in *Pds5a*^{GT} WT and *Pds5a*^{GT} KO ESCs. For each plot, normalized read density is plotted in 10 kb window centred around the TSS. **b,** Boxplots compare PcG protein and histone modification cChIP-seq signal at all vs. upregulated PRC1/PRC2 target genes (left) or at all vs. upregulated vPRC1 target genes in wild-type and *Pds5a*^{GT} KO ESCs. Shown are median (horizontal line), 25th to 75th percentiles (boxes), and 90% (whiskers). Significance was determined by Wilcoxon rank-sum test. Asterisks indicate significant differences between groups (* $p < 0.01$; ** $p < 0.001$; *** $p < 0.0001$; **** $p < 0.00001$; n.s. – not significant). **c,** ChIP-qPCR compares PcG proteins and histone modifications at selected PRC1/PRC2 target genes in wild-type and *Pds5a* KO ESCs. Shown are data of two independent experimental replicates. IAP serves as negative control. Source data are provided as a Source Data file. **d,** Box plots compare ATAC-seq signals in *Pds5a*^{GT} WT and *Pds5a*^{GT} KO ESCs at all vs. significantly upregulated PRC1/PRC2 target genes or at all vs. significantly upregulated vPRC1 target genes. Shown are median (horizontal line), 25th to 75th percentiles (boxes), and 90% (whiskers). Significance was determined by Wilcoxon rank-sum test. Asterisks indicate significant differences between groups (* $p < 0.01$; ** $p < 0.001$; *** $p < 0.0001$; **** $p < 0.00001$; n.s. – not significant).

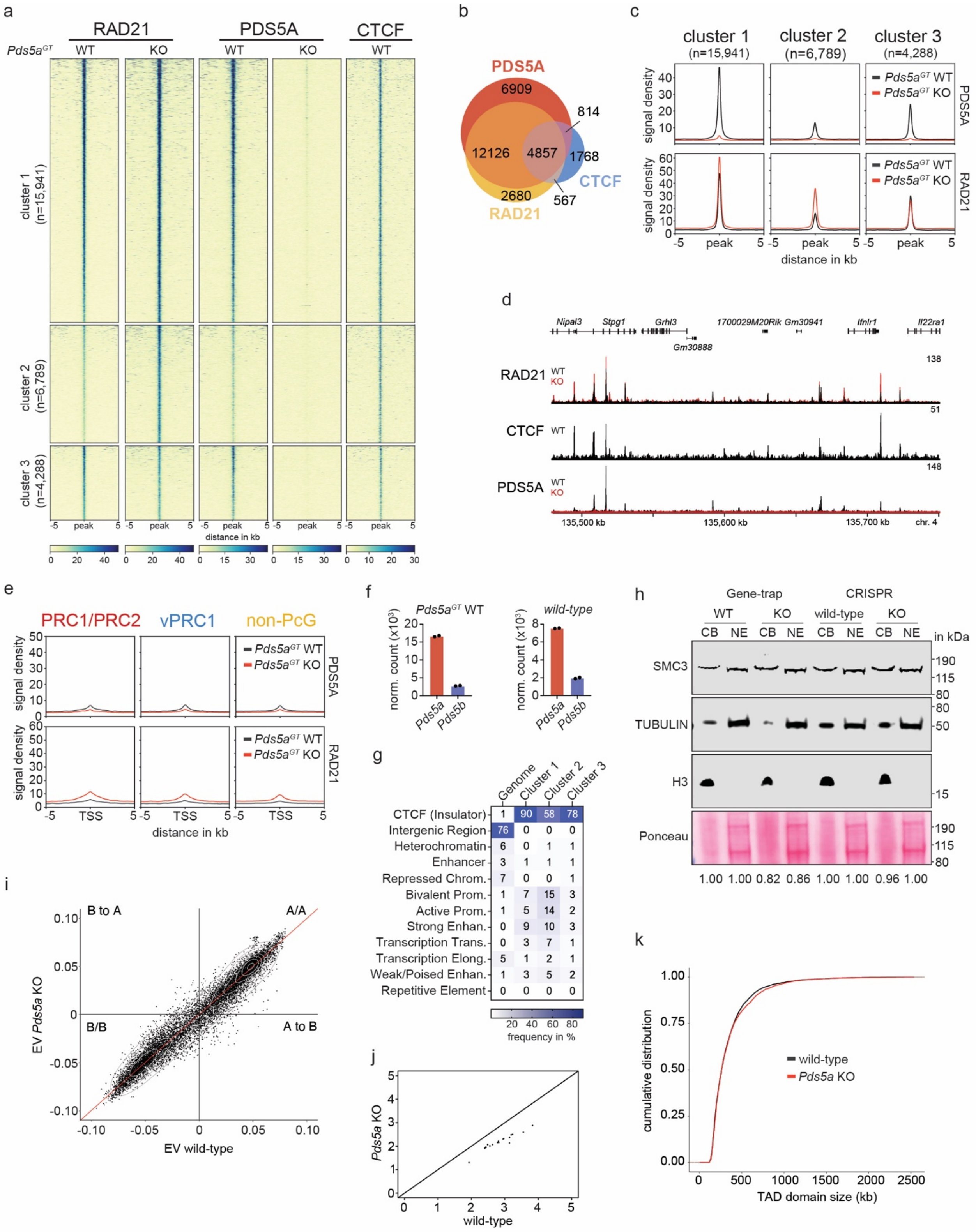

a
RAD21
PDS5A
CTCF
Pds5a^GT
WT
KO
WT
KO
WT
cluster 1 (n=15,941)
cluster 2 (n=6,789)
cluster 3 (n=4,288)
-5 peak 5
distance in kb
b
PDS5A
6909
814
12126
4857
1768
CTCF
2680
567
RAD21
c
cluster 1 (n=15,941)
cluster 2 (n=6,789)
cluster 3 (n=4,288)
signal density
Pds5a^GT WT
Pds5a^GT KO
PDS5A
RAD21
distance in kb
d
Nipal3
Stpg1
Grhl3
1700029M20Rik
Gm30941
Ifnlr1
Il22ra1
Gm30888
RAD21
CTCF
PDS5A
WT
KO
138
51
148
135,500 kb
135,600 kb
135,700 kb
chr. 4
e
PRC1/PRC2
vPRC1
non-PcG
signal density
Pds5a^GT WT
Pds5a^GT KO
PDS5A
RAD21
TSS
distance in kb
f
Pds5a^GT WT
wild-type
norm. count (x10^3)
Pds5a
Pds5b
g
Genome
Cluster 1
Cluster 2
Cluster 3
CTCF (Insulator)
Intergenic Region
Heterochromatin
Enhancer
Repressed Chrom.
Bivalent Prom.
Active Prom.
Strong Enhan.
Transcription Trans.
Transcription Elong.
Weak/Poised Enhan.
Repetitive Element
frequency in %
h
Gene-trap
CRISPR
WT
KO
wild-type
KO
CB
NE
in kDa
SMC3
TUBULIN
H3
Ponceau
1.00 1.00 0.82 0.86 1.00 1.00 0.96 1.00
i
B to A
A/A
B/B
A to B
EV Pds5a KO
EV wild-type
j
Pds5a KO
wild-type
k
cumulative distribution
wild-type
Pds5a KO
TAD domain size (kb)

Supplementary Fig. 4. PDS5A colocalizes with cohesin and stabilizes chromatin binding. a, cChIP-seq heatmaps of RAD21 and PDS5A signals in *Pds5a*^{GT} WT and *Pds5a*^{GT} KO ESCs. Enrichment signal is plotted +/- 5 kb around RAD21 peaks and grouped into three clusters. **b,** Euler diagrams show overlap of CTCF, RAD21 and PDS5A peaks in *Pds5a*^{GT} WT ESCs. **c,** Meta plots show average PDS5A and RAD21 cChIP-seq signals in clusters 1-3 in *Pds5a*^{GT} WT and *Pds5a*^{GT} KO ESCs. For each plot, normalized read density is plotted in 10 kb window centered around RAD21 peaks. **d,** Genomic screenshot of RAD21 and PDS5A cChIP-seq signals in *Pds5a*^{GT} WT (black) and *Pds5a*^{GT} KO ESCs and of CTCF ChIP-seq in *Pds5a*^{GT} WT ESCs. **e,** Meta plots show average PDS5A and RAD21 cChIP-seq signals at PRC1/PRC2, vPRC1 and non-PcG target genes in *Pds5a*^{GT} WT and *Pds5a*^{GT} KO ESCs. For each plot, normalized read density is plotted in 10 kb window centered around the TSS. **f,** RNA-seq normalized read counts of *Pds5a* and *Pds5b* in *Pds5a*^{GT} WT and wild-type ESCs. Shown are mean of two experimental replicates (dots depict replicates). **g,** Heatmap of genomic annotation frequency of RAD21 peaks in the three clusters based on ChromHMM. Genome serves as reference. Numbers within the fields indicate % observation frequency. **h,** Western blot analysis of SMC3, TUBULIN and H3 signals in nuclear soluble extract (NE) and chromatin-bound (CB) fraction of *Pds5a*^{GT} WT and *Pds5a*^{GT} KO ESCs, and wild-type and *Pds5a* KO ESCs. Numbers below indicate SMC3 signal normalized by Ponceau staining. **i,** Scatter plot of compartment signal (Eigenvector) for each 250 kb bin in the genome in wild-type vs. *Pds5a* KO ESCs. **j,** Scatter plot of compartment strength for each chromosome (dot) in wild-type vs. *Pds5a* KO ESCs. **k,** Cumulative distributions of called TAD domains in wild-type vs. *Pds5a* KO ESCs.

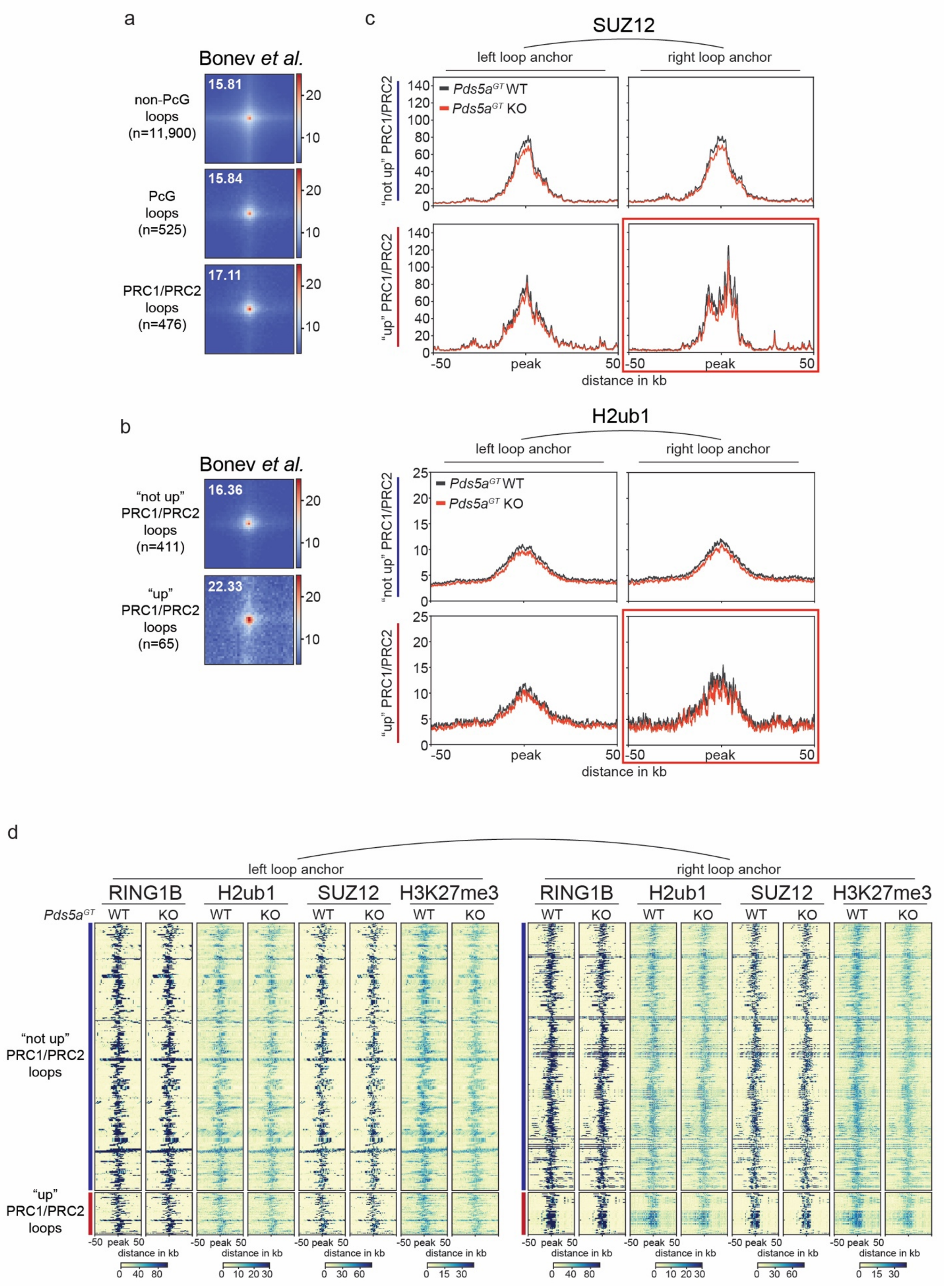
a
Bonev et al.
non-PcG loops (n=11,900)
15.81
PcG loops (n=525)
15.84
PRC1/PRC2 loops (n=476)
17.11
b
Bonev et al.
"not up" PRC1/PRC2 loops (n=411)
16.36
"up" PRC1/PRC2 loops (n=65)
22.33
c
SUZ12
left loop anchor
right loop anchor
Pds5a^GT WT
Pds5a^GT KO
"not up" PRC1/PRC2
"up" PRC1/PRC2
-50
peak
50
distance in kb
H2ub1
left loop anchor
right loop anchor
Pds5a^GT WT
Pds5a^GT KO
"not up" PRC1/PRC2
"up" PRC1/PRC2
distance in kb
d
left loop anchor
right loop anchor
RING1B
H2ub1
SUZ12
H3K27me3
Pds5a^GT
WT
KO
"not up" PRC1/PRC2 loops
"up" PRC1/PRC2 loops
-50 peak 50
distance in kb

Supplementary Fig. 5. PDS5A is required for maintenance of repressive Polycomb loops crossing ultra-long distances. a, Loop pileup analysis of non-PcG loops (n=11,900), PcG loops (PRC1/2 and vPRC1, n=525) and PRC1/PRC2 loops (PRC1/2 only, n=476) using published HiC data [65] in ESCs. Number indicates relative peak enrichment. **b,** Loop pileup analysis of Polycomb loops overlapping unchanged/downregulated PRC1/PRC2 target genes (not up PRC1/PRC2 loop; n=411) and upregulated PRC1/PRC2 target genes (up PRC1/PRC2 loop; n=65) upon PDS5A deletion using published HiC data [65] in ESCs. Number indicates relative peak enrichment. **c,** Meta plots show average SUZ12 and H2Aub1 cChIP-seq signals at Polycomb loop anchors associated with unchanged/downregulated PRC1/PRC2 target genes (not up PRC1/PRC2 loop; n=411) and upregulated PRC1/PRC2 target genes (up PRC1/PRC2 loop; n=65) in wild-type (left) and *Pds5a* KO (right) ESCs. Red box indicates loop anchor that overlaps upregulated PRC1/PRC2 target genes in *Pds5a* KO ESCs. **d,** cChIP-seq heatmaps of RING1B, H2Aub1, SUZ12 and H3K27me3 at Polycomb loops between PRC1/PRC2 genes. Heatmaps are divided in Polycomb loops between unchanged/downregulated PRC1/PRC2 target genes (not up PRC1/PRC2 loop; n=411) and upregulated PRC1/PRC2 target genes (up PRC1/PRC2 loop; n=65).

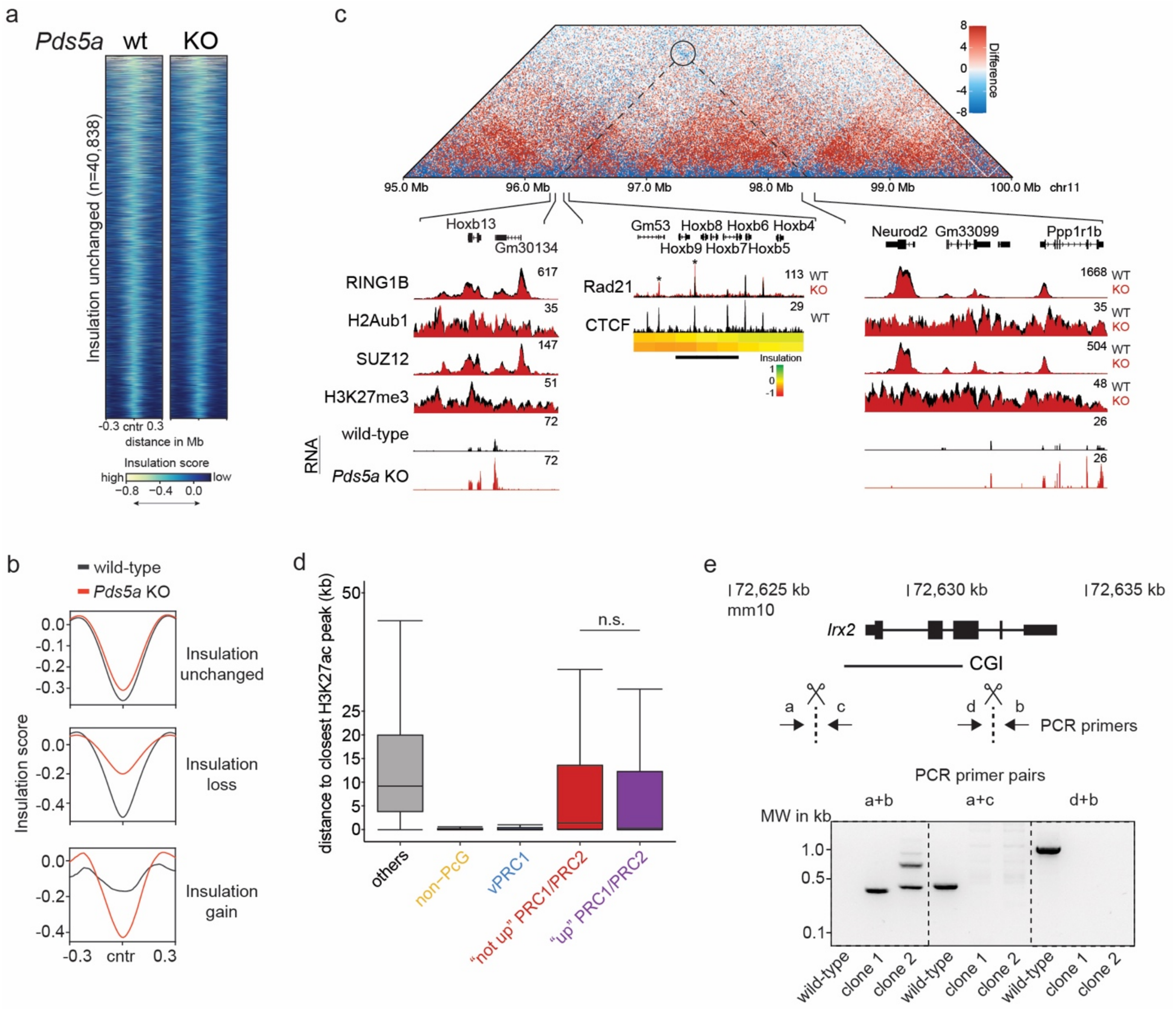

Supplementary Fig. 6. Loss of repressive Polycomb loops is linked to cohesin-mediated insulation gain. a, Heatmaps show insulation scores in wild-type and *Pds5a* KO ESCs for unchanged insulated regions (n=40,838) upon PDS5A deletion. Insulation scores are plotted +/- 300 kb around insulated regions. **b,** Meta plots show average insulation scores in wild-type and *Pds5a* KO ESCs for insulated regions that were unchanged, were lost or gained upon PDS5A deletion. For each plot, insulation scores are plotted in 600 kb window centred around insulated regions. **c,** Top: HiC matrix shows interaction differences on chromosome 11 between wild-type and *Pds5a* KO ESCs (blue = loss; red = gain). Bottom: Genomic screenshots of cChIP-seq of PcG proteins and histone modifications and of normalized RNA-seq counts at *Hoxb13* and *Neurod2* in wild-type (black) and *Pds5a* KO (red) ESCs. RAD21 and CTCF cChIP-seq signals and insulation score heatmap are shown at the *Hoxb* gene cluster, the insulation-gaining region (indicated by black bar). **d,** Boxplots shows genomic distances of gene class TSSs to closest H3K27ac peaks. Shown are median (horizontal line), 25th to 75th percentiles (boxes), and 90% (whiskers). Significance was determined by Wilcoxon rank-sum test. (n.s. – not significant). **e,** CRISPR design, genotyping strategy and PCR validation of Polycomb loop anchor excision at *Irx2*.

Supplementary Table 1
CRISPR screen results with gene name, Mageck RRA score, relative enrichment and rank for hits with p-value <0.01.

Gene name	Mageck RRA score	$-Log_{10}$ (Mageck RRA score)	p-value	rank	log2 FC
Trp53	1.10E-11	10.96	7.44E-07	1	0.58
Pds5a	3.03E-09	8.52	7.44E-07	2	3.27
Cbx7	1.79E-07	6.75	7.44E-07	3	1.54
Zic3	1.56E-06	5.81	2.23E-06	4	1.86
Hist3h2a	4.28E-06	5.37	3.50E-05	5	-3.09
Ezh2	1.35E-05	4.87	5.43E-05	6	1.82
Suz12	1.43E-05	4.85	6.02E-05	7	2.54
Morc2a	1.84E-05	4.73	7.66E-05	8	1.85
Pax5	2.27E-05	4.64	2.16E-04	9	-0.59
Actr8	2.51E-05	4.60	1.88E-04	10	0.56
Carm1	3.16E-05	4.50	2.27E-04	11	-1.17
Casp2	3.84E-05	4.42	1.57E-04	12	-5.64
Fam175a	4.66E-05	4.33	3.46E-04	13	-0.81
Jmjd1c	4.70E-05	4.33	3.47E-04	14	-0.90
Eed	5.54E-05	4.26	2.28E-04	15	1.94
Pmf1	7.17E-05	4.14	5.04E-04	16	0.65
Pola2	9.98E-05	4.00	6.94E-04	17	-2.03
Zfp451	1.15E-04	3.94	4.48E-04	18	-3.52
Rnf8	1.21E-04	3.92	7.58E-04	19	-1.77
Gcat	1.25E-04	3.90	4.87E-04	20	2.03
Cacna1d	1.32E-04	3.88	8.80E-04	21	0.02
Numa1	1.35E-04	3.87	5.45E-04	22	1.16
Xrcc4	1.52E-04	3.82	9.96E-04	23	-1.59
Nup205	1.78E-04	3.75	6.89E-04	24	2.03
Psmc1	1.92E-04	3.72	7.50E-04	25	-1.43
Mphosph8	2.28E-04	3.64	8.89E-04	26	1.50
Taf10	2.91E-04	3.54	1.70E-03	27	-2.21
Gm5382	3.25E-04	3.49	1.26E-03	28	1.71
Actl6a	3.39E-04	3.47	2.44E-03	29	-2.70
Rnf2	3.60E-04	3.44	1.39E-03	30	1.09
Stat1	3.76E-04	3.42	2.38E-03	31	0.00
St18	3.86E-04	3.41	1.50E-03	32	-0.35
Nr3c2	3.92E-04	3.41	2.46E-03	33	-1.60
Tiparp	4.36E-04	3.36	1.66E-03	34	1.46
Lsm6	4.60E-04	3.34	1.75E-03	35	0.89
Rrp12	4.60E-04	3.34	1.75E-03	36	0.38
Foxp3	4.68E-04	3.33	2.80E-03	37	0.57
Cdk4	5.19E-04	3.29	3.13E-03	38	-1.61
Cenpp	6.53E-04	3.18	2.48E-03	39	-1.35
Chd1	6.71E-04	3.17	3.92E-03	40	-1.84
Nfe2l2	6.97E-04	3.16	4.05E-03	41	-2.36

Elavl1	6.97E-04	3.16	2.65E-03	42	0.07
Setd2	6.97E-04	3.16	4.08E-03	43	-2.70
Taf1	8.04E-04	3.09	4.61E-03	44	-1.61
Gatad2b	8.81E-04	3.05	5.12E-03	45	-2.63
Cacna1b	8.98E-04	3.05	4.01E-03	46	-0.26
Ptpn4	1.04E-03	2.98	4.67E-03	47	-1.40
Ciao1	1.07E-03	2.97	2.87E-03	48	2.01
Mdm2	1.07E-03	2.97	6.08E-03	49	-2.36
Dr1	1.07E-03	2.97	3.89E-03	50	-0.10
Taf1c	1.07E-03	2.97	3.89E-03	51	-0.01
Ep300	1.11E-03	2.96	6.29E-03	52	-0.69
Cacng1	1.11E-03	2.96	4.96E-03	53	0.36
Ncor1	1.16E-03	2.94	6.55E-03	54	-0.86
Tsnax	1.20E-03	2.92	4.37E-03	55	0.17
Adig	1.42E-03	2.85	5.10E-03	56	-4.06
Limk2	1.46E-03	2.84	5.22E-03	57	-1.72

Supplementary Table 2
Next Generation Sequencing statistics of ChIP-seq, RNA-seq and HiC.

RNA-seq statistics

Sample name	# million filtered reads uniquely mapped to the mouse genome (mm10)	Sequencing mode
Genetrap_wt_rep1	25.5	PE150
Genetrap_wt_rep2	25	PE150
Genetrap_Pds5a_KO_rep1	38.1	PE150
Genetrap_Pds5a_KO_rep2	35.8	PE150
wt_rep1	21	SE50
wt_rep2	18.3	SE50
Pds5a_KO_rep1	18	SE50
Pds5a_KO_rep2	19.2	SE50

cChIP-seq statistics

Sample name	# of total uniquely mapped reads	# of filtered reads uniquely mapped to the mouse genome (mm10)	# of filtered reads uniquely mapped to the human genome (hg38)	Sequencing mode
Input_wt	30,180,572	28,241,510	1,939,062	PE150
Input_Pds5a_KO	26,672,594	24,930,198	1,742,396	PE150
H2Aub_wt_rep1	24,571,504	19,876,190	4,695,314	PE150
H2Aub_wt_rep2	27,577,968	22,294,808	5,283,160	PE150
H2Aub_Pds5a_KO_rep1	27,462,904	21,804,616	5,658,288	PE150
H2Aub_Pds5a_KO_rep2	32,058,518	25,450,838	6,607,680	PE150
K27me3_wt_rep1	47,795,516	45,094,916	2,700,600	PE150
K27me3_wt_rep2	28,411,706	27,086,766	1,324,940	PE150
K27me3_Pds5a_KO_rep1	49,772,922	46,892,754	2,880,168	PE150
K27me3_Pds5a_KO_rep2	39,035,030	37,157,254	1,877,776	PE150
Rnf2_wt_rep1	54,153,586	52,792,902	1,360,684	PE150
Rnf2_wt_rep2	49,681,830	48,052,050	1,629,780	PE150
Rnf2_Pds5a_KO_rep1	38,368,316	36,808,574	1,559,742	PE150
Rnf2_Pds5a_KO_rep2	42,130,506	40,539,128	1,591,378	PE150
Cbx7_wt_rep1	28,762,882	27,189,198	1,573,684	PE150
Cbx7_wt_rep2	27,874,380	26,471,838	1,402,542	PE150
Input_wt	53,377,272	48,446,568	4,930,704	PE150
Input_Pds5a_KO	48,292,340	43,916,472	4,375,868	PE150
Pcgf1_wt_rep1	27,490,912	24,411,170	3,079,742	PE150
Pcgf1_wt_rep2	17,250,462	15,202,874	2,047,588	PE150

CTCF_wt_rep1	18,644,566	16,923,070	1,721,496	PE150
CTCF_wt_rep2	30,558,054	27,787,302	2,770,752	PE150
Rad21_wt_rep1	28,215,532	25,093,976	3,121,556	PE150
Rad21_wt_rep2	26,980,128	23,767,182	3,212,946	PE150
Rad21_Pds5a_KO_rep1	31,529,766	28,809,560	2,720,206	PE150
Rad21_Pds5a_KO_rep2	30,020,762	27,467,002	2,553,760	PE150
Suz12_wt_rep1	20,913,958	19,949,384	964,574	PE150
Suz12_wt_rep2	22,088,794	21,060,210	1,028,584	PE150
Suz12_Pds5a_KO_rep1	25,144,526	23,596,050	1,548,476	PE150
Suz12_Pds5a_KO_rep2	26,569,506	25,568,826	1,000,680	PE150

Hi-C statistics

**Sample name**	**NGS Library name**	**Sequencing mode**	**# Filtered uniquely mapped contacts/ library**	**# Filtered uniquely mapped contacts/ replicate**	**# Total filtered uniquely mapped contacts/ genotype**
HiC_wt_rep1	HiC_wt_rep1_1	PE150	3,517,962	7,223,256	365,265,758
	HiC_wt_rep1_2	PE150	3,705,294		
HiC_wt_rep2	HiC_wt_rep2_1	PE150	71,632,962	71,632,962	
HiC_wt_rep3	HiC_wt_rep3_1	PE150	6,296,696	33,600,346	
	HiC_wt_rep3_2	PE150	5,610,322		
	HiC_wt_rep3_3	PE150	5,971,198		
	HiC_wt_rep3_4	PE150	4,748,521		
	HiC_wt_rep3_5	PE150	5,136,263		
	HiC_wt_rep3_6	PE150	5,837,346		
HiC_wt_rep4	HiC_wt_rep4_1	PE150	17,692,550	91,946,962	
	HiC_wt_rep4_2	PE150	15,516,821		
	HiC_wt_rep4_3	PE150	16,558,075		
	HiC_wt_rep4_4	PE150	12,782,701		
	HiC_wt_rep4_5	PE150	14,153,652		
	HiC_wt_rep4_6	PE150	15,243,163		
HiC_wt_rep5	HiC_wt_rep5_1	PE150	35,805,298	160,862,232	
	HiC_wt_rep5_2	PE150	31,228,863		
	HiC_wt_rep5_3	PE150	33,513,149		
	HiC_wt_rep5_5	PE150	29,096,861		
	HiC_wt_rep5_6	PE150	31,218,061		
HiC_Pds5a_KO_rep1	HiC_Pds5a_KO_rep1_1	PE150	3,443,297	6,082,589	363,748,431
	HiC_Pds5a_KO_rep1_2	PE150	2,639,292		
HiC_Pds5a_KO_rep2	HiC_Pds5a_KO_rep2_1	PE150	39,922,068	72,404,203	

	HiC_Pds5a_KO_rep2_2	PE150	32,482,135		
HiC_Pds5a_KO_rep3	HiC_Pds5a_KO_rep3_1	PE150	3,532,882	27,599,484	
	HiC_Pds5a_KO_rep3_2	PE150	3,553,986		
	HiC_Pds5a_KO_rep3_3	PE150	4,034,161		
	HiC_Pds5a_KO_rep3_4	PE150	6,209,504		
	HiC_Pds5a_KO_rep3_5	PE150	5,907,008		
	HiC_Pds5a_KO_rep3_6	PE150	4,361,943		
HiC_Pds5a_KO_rep4	HiC_Pds5a_KO_rep4_1	PE150	13,338,588	84,106,810	
	HiC_Pds5a_KO_rep4_2	PE150	13,470,350		
	HiC_Pds5a_KO_rep4_3	PE150	14,907,731		
	HiC_Pds5a_KO_rep4_4	PE150	15,571,926		
	HiC_Pds5a_KO_rep4_5	PE150	15,560,001		
	HiC_Pds5a_KO_rep4_6	PE150	11,258,214		
HiC_Pds5a_KO_rep5	HiC_Pds5a_KO_rep5_1	PE150	27,381,604	173,555,345	
	HiC_Pds5a_KO_rep5_2	PE150	27,707,816		
	HiC_Pds5a_KO_rep5_3	PE150	30,576,807		
	HiC_Pds5a_KO_rep5_4	PE150	32,278,209		
	HiC_Pds5a_KO_rep5_5	PE150	32,281,960		
	HiC_Pds5a_KO_rep5_6	PE150	23,328,949		

Supplementary Table 3
List of coordinates (mm9 reference genome) and genomic screenshots of 25 Polycomb and non-Polycomb target loci profiled by ChIP-CapSeq. Genomic screenshots show enrichments of PcG proteins and histone modifications before (black) and after ectopic TetR-CBX7 expression (orange).

Dazl locus
chr17: 50,414,245-50,444,405

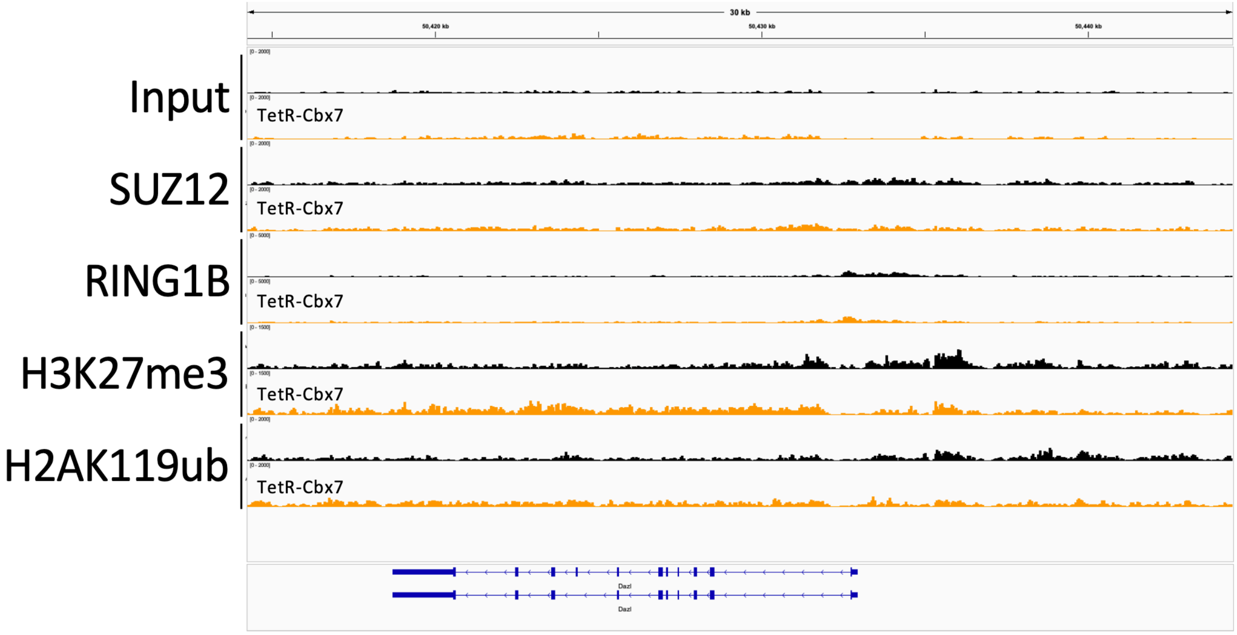

Oct4/Pouf51 locus
chr17:35623948-35666758

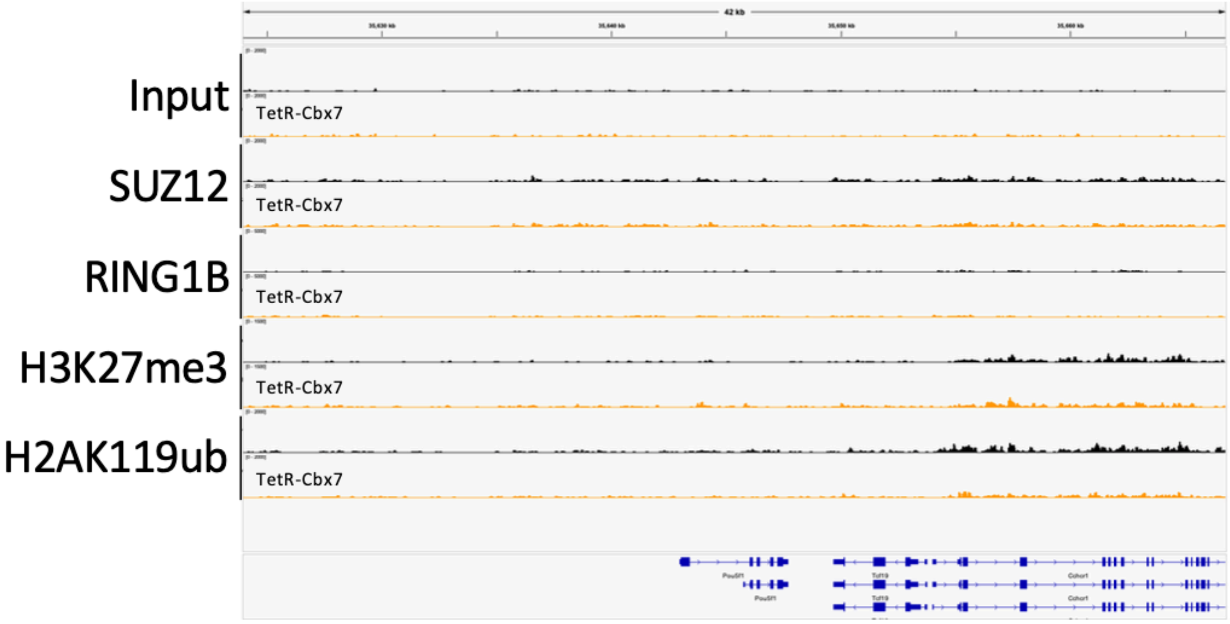

Olig1 & *Olig2* locus
chr16: 91,199,329-91,300,463

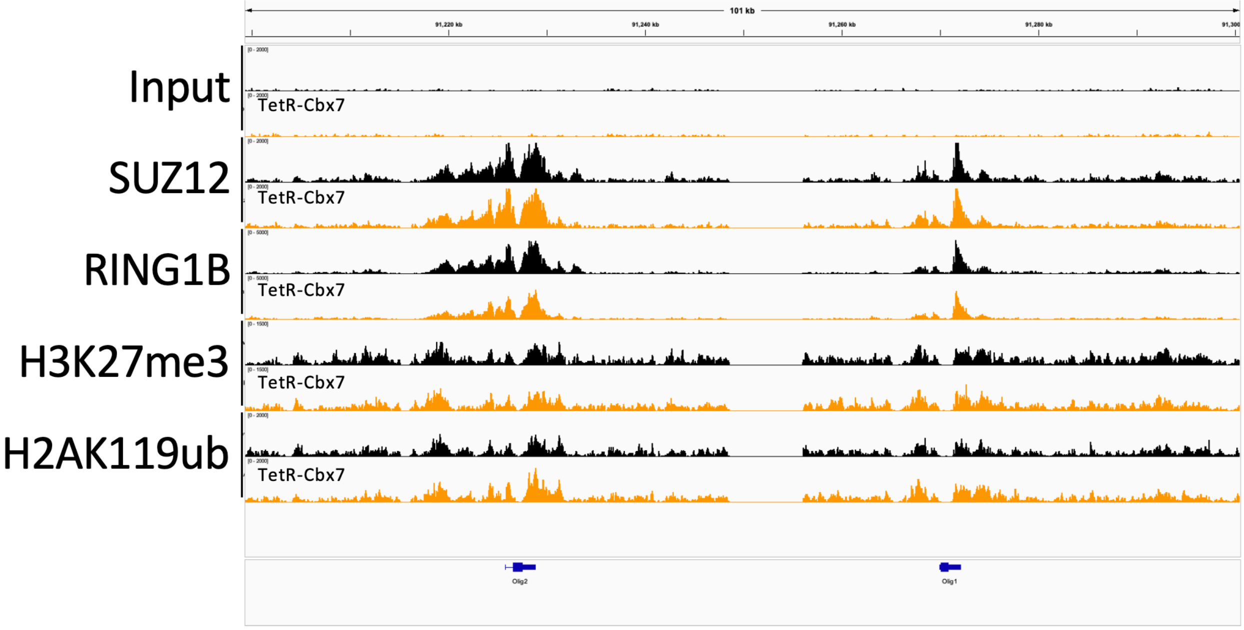

Sox17 locus
chr1: 4460681-4511256

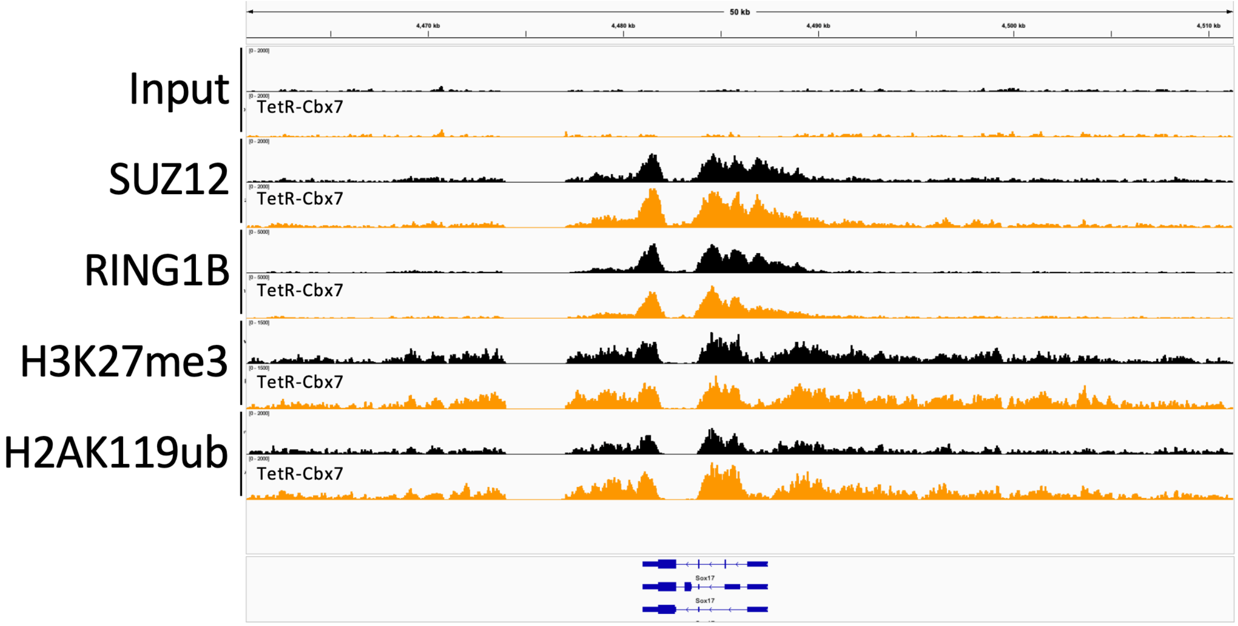

Intergenic *Crisp4* locus
chr1:17,995,977-18,046,157

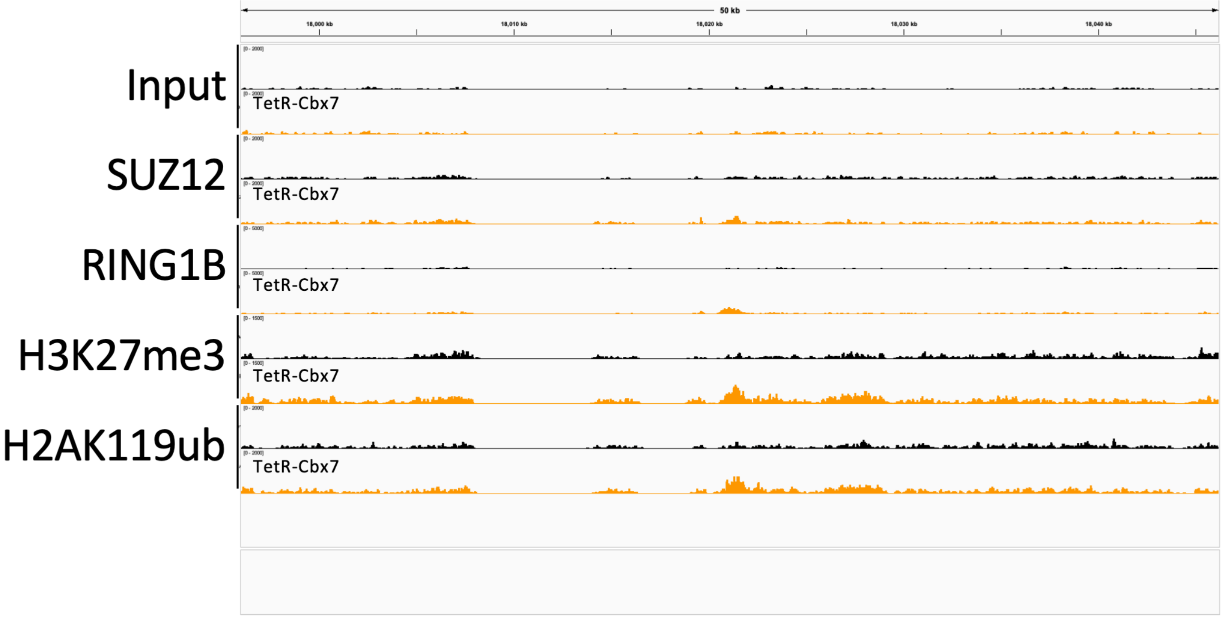

Hoxd gene cluster
chr2:74,450,084-74,651,064

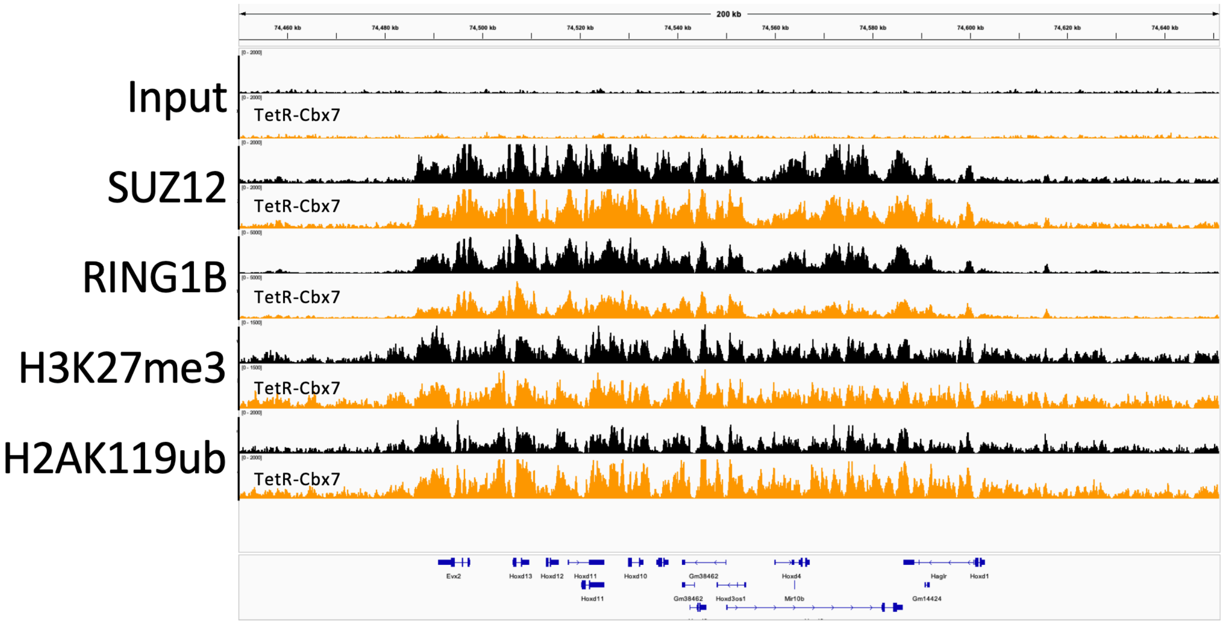

Pax6 locus
chr2:105,488,775-105,539,252

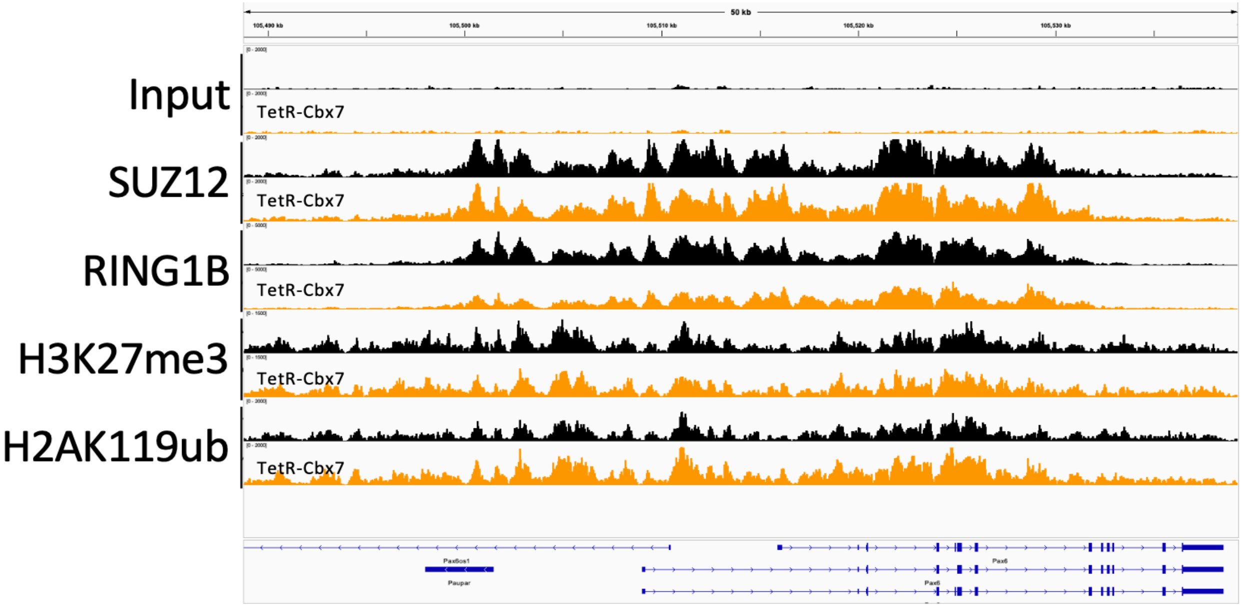

Commd7 & Dnmt3b locus
chr2:153432137-153522909

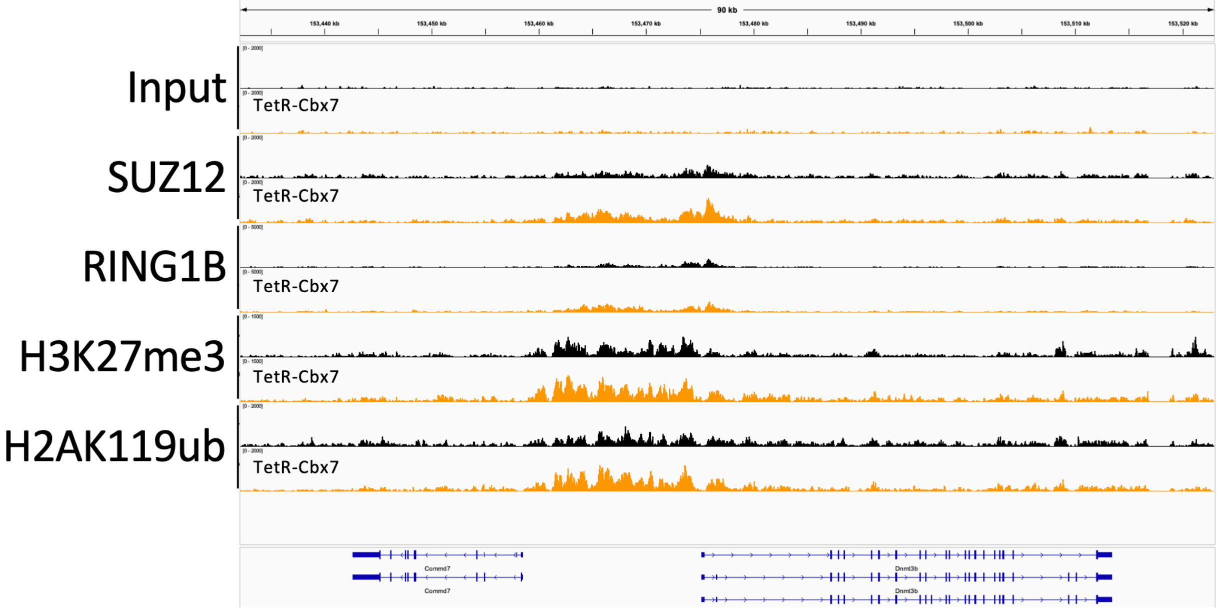

Sox2 locus
chr3:34,537,459-34,568,988

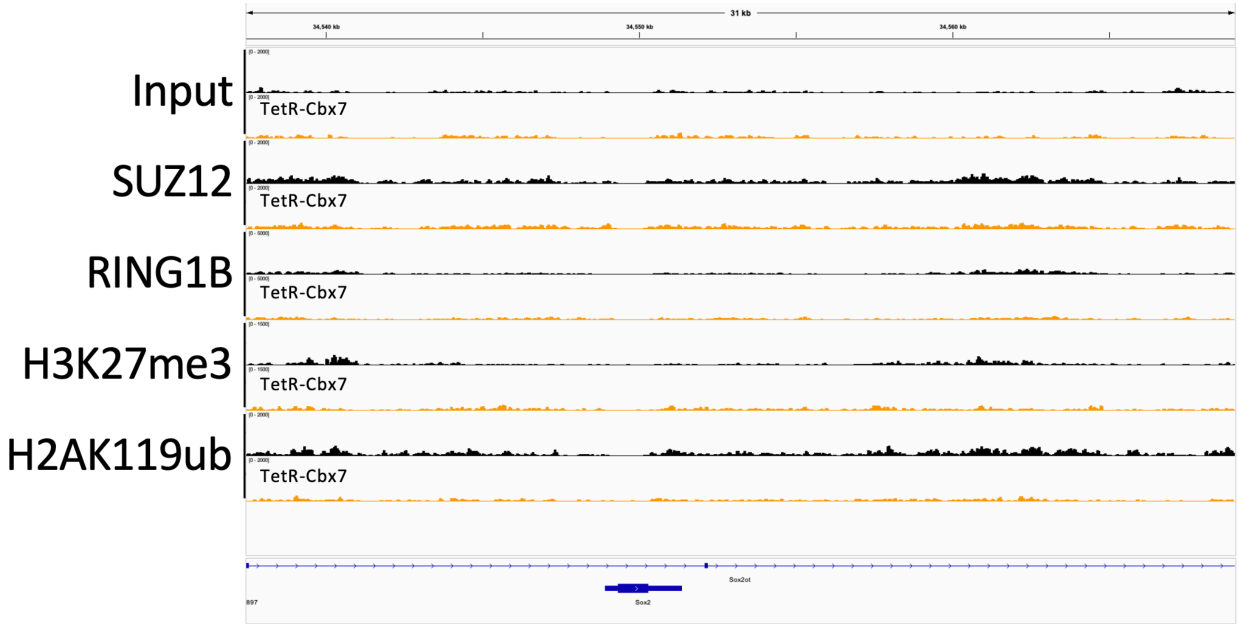

Mef2d locus
chr3:87,931,198-88,031,358

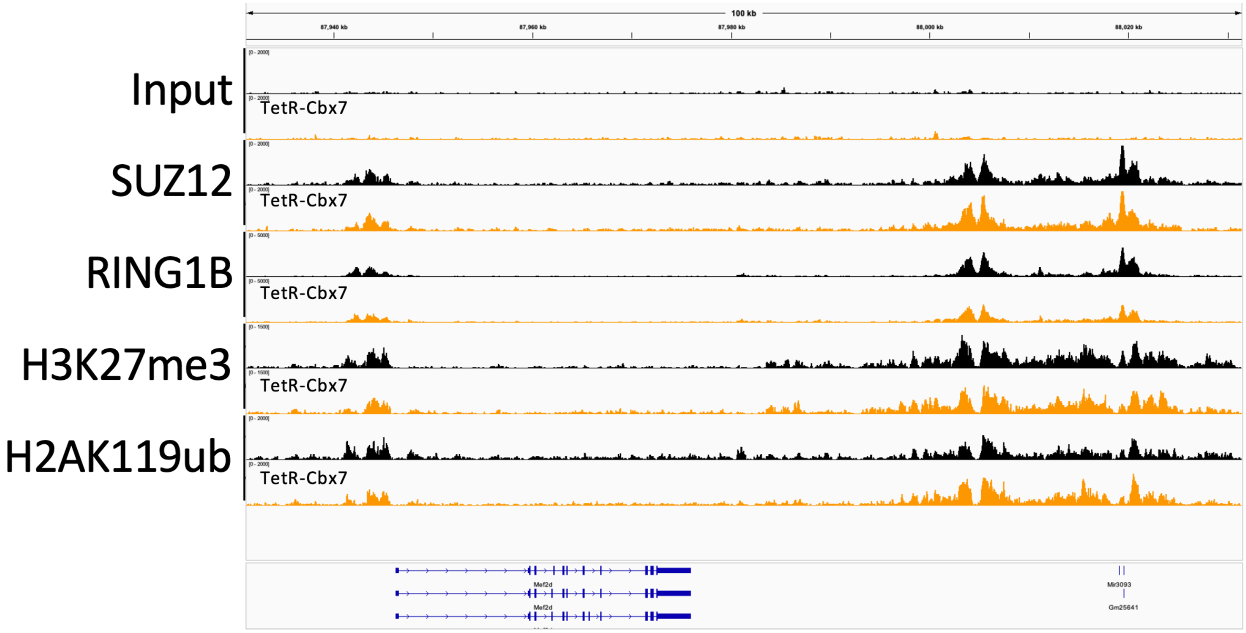

Neurog2 locus
chr3:127,332,802-127,342,995

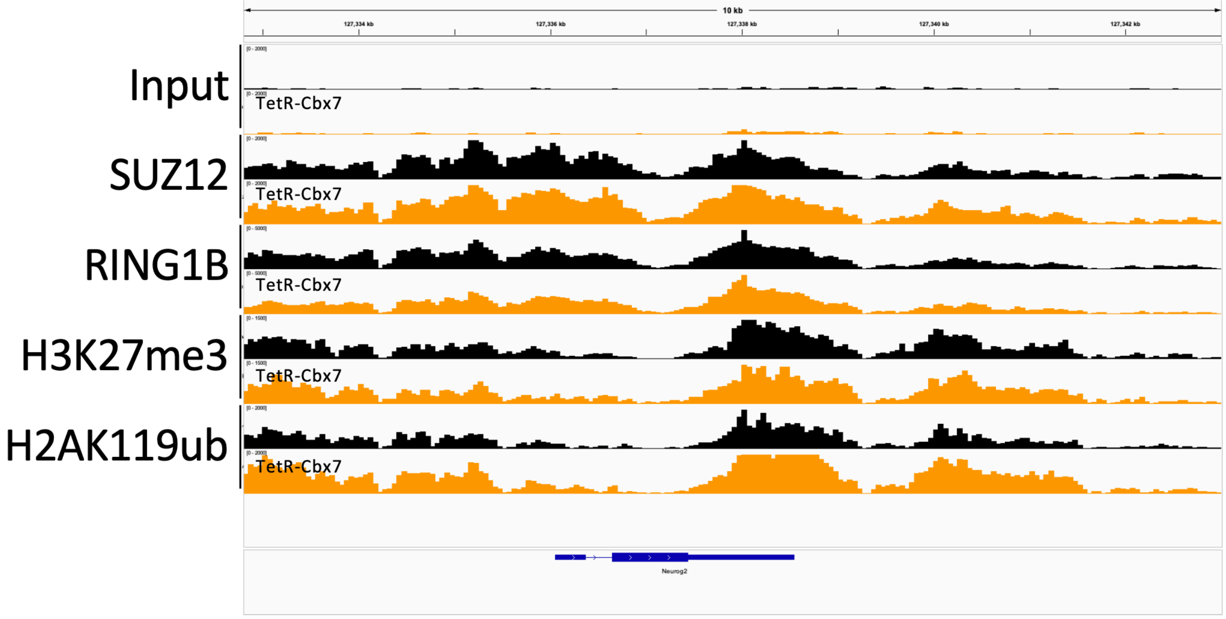

Cdkn2a & b & Gm12610 locus
chr4:88905858-88962573

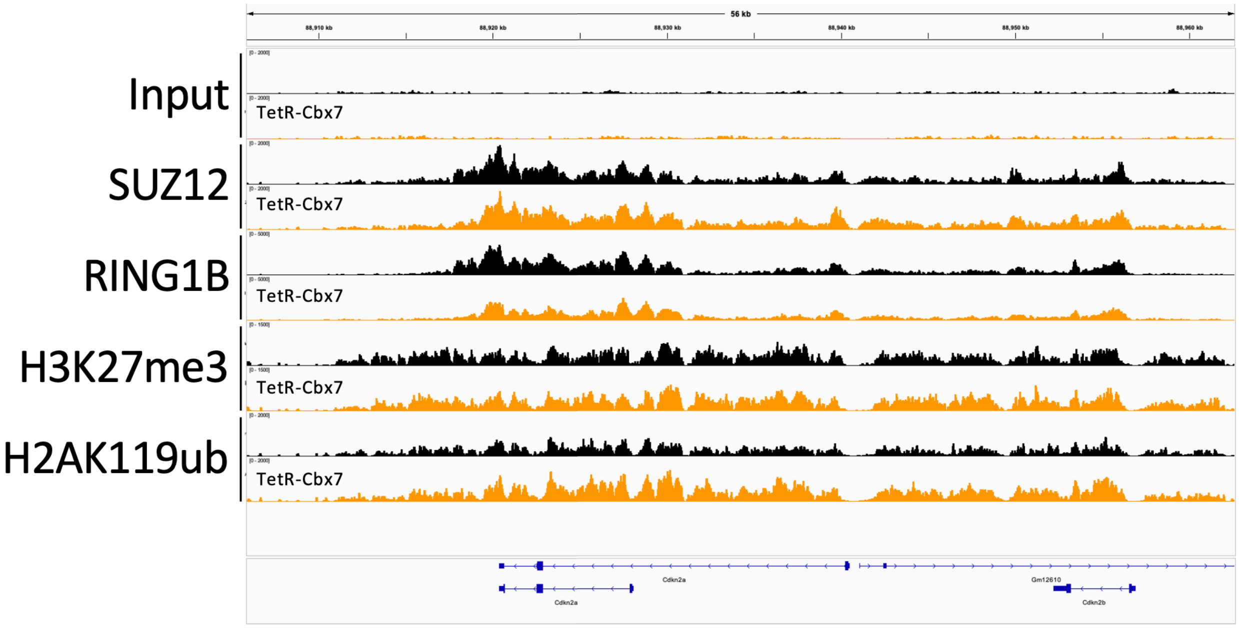

Pou3f1 locus
chr4:124294578-124395096

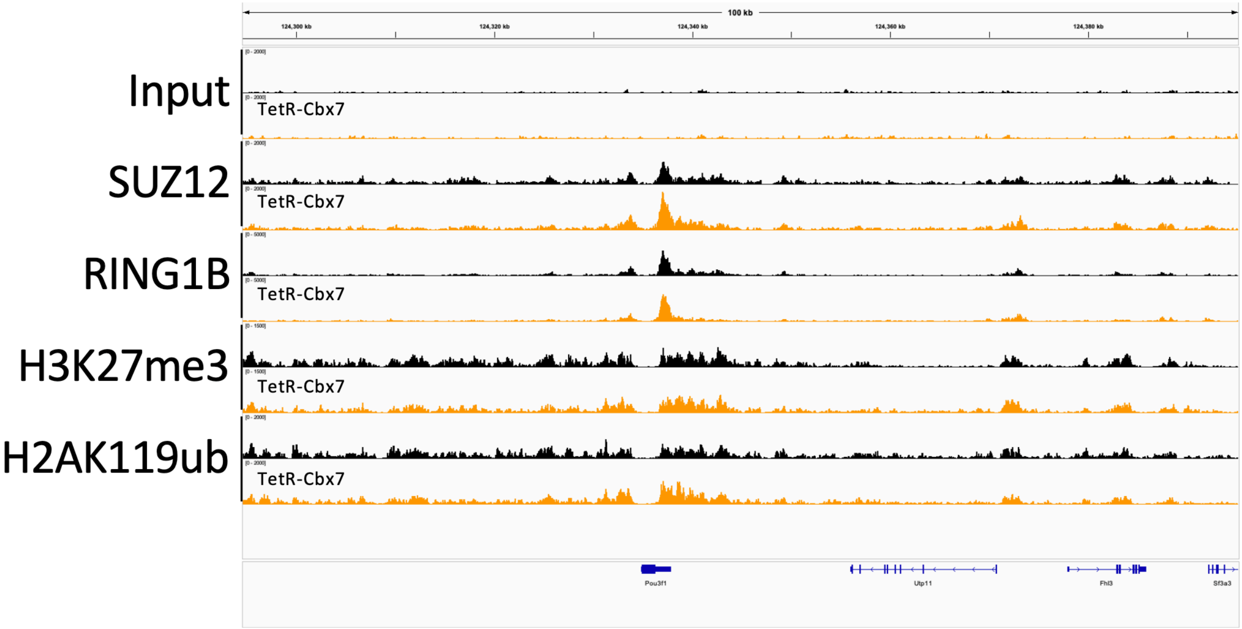

Prdm8 & Fgf5 & 1700007G11Rik locus
chr5:98,563,339-98,814,815

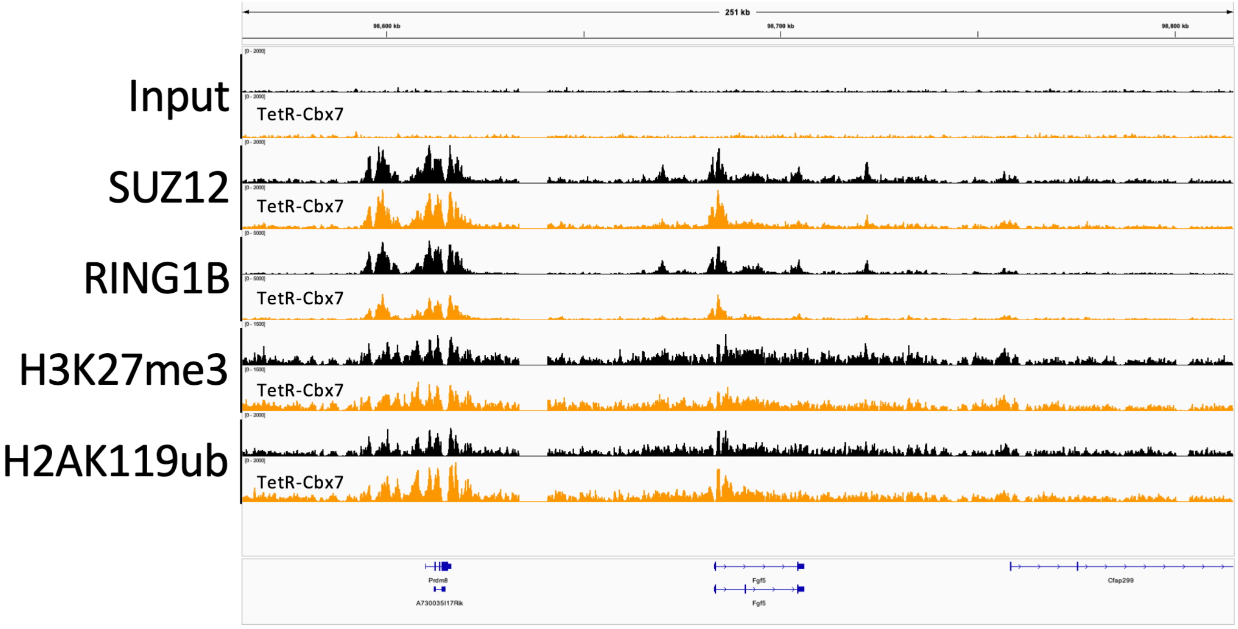

Tbx3 locus
chr5:120,094,791-120,194,420

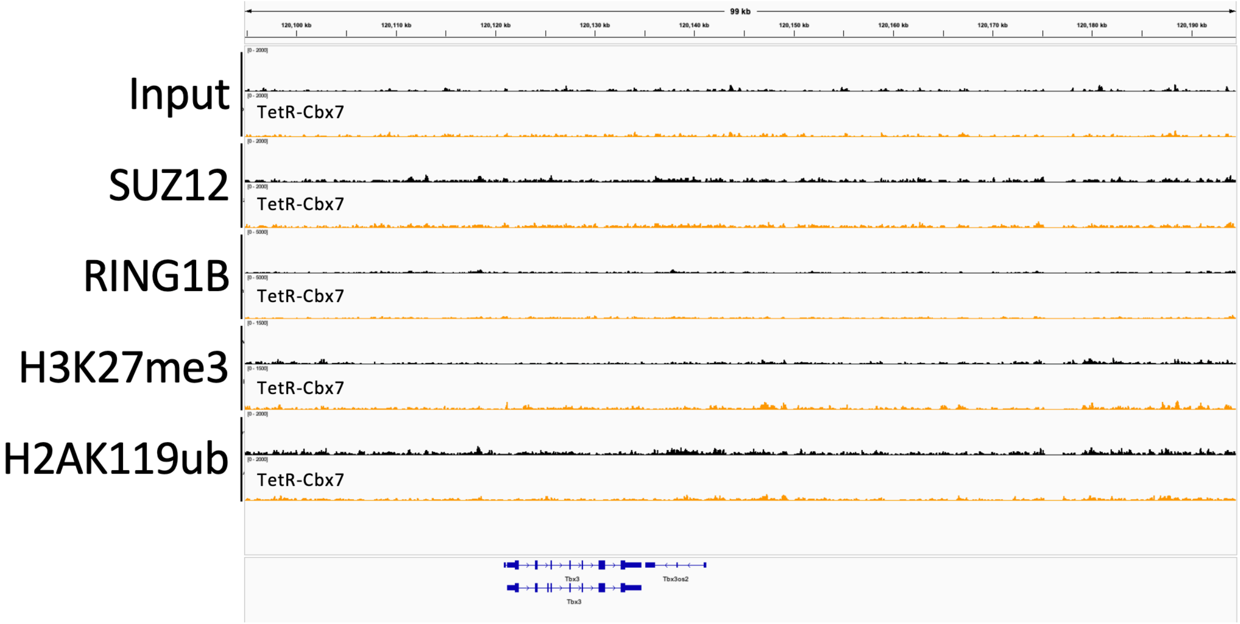

Nanog locus
chr6:122,642,090-122,677,539

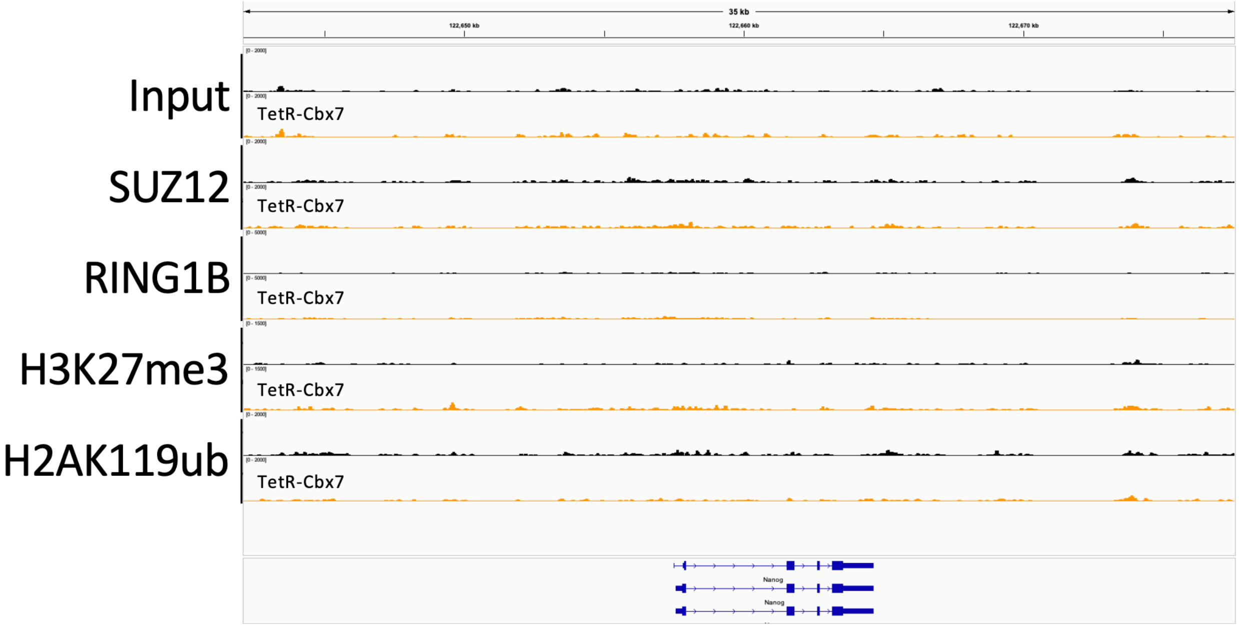

Hbb gene cluster locus
chr7:110,957,144-111,008,233

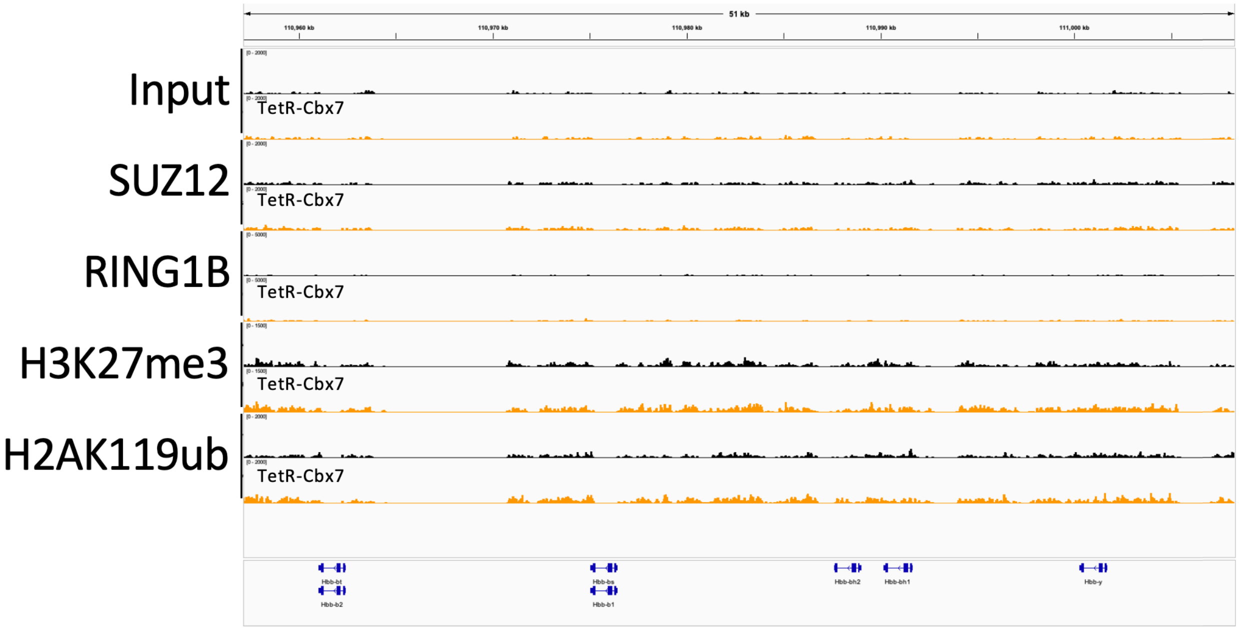

Plekha1 & *Htra1* locus
chr7:137,983,359-138,081,333

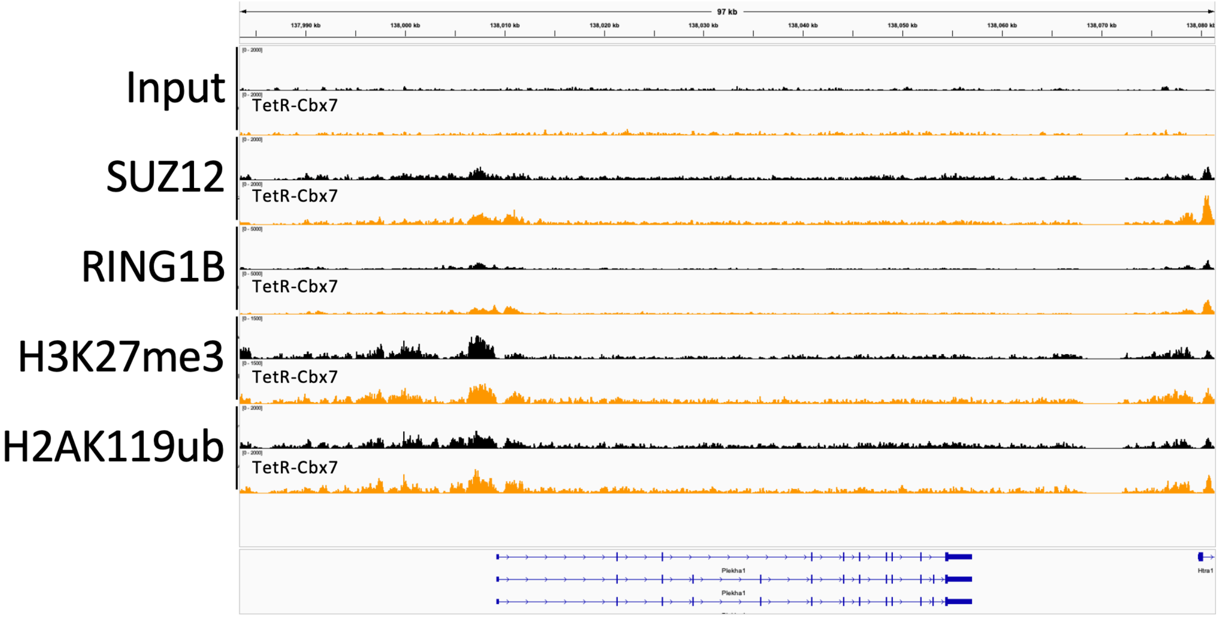

Igf2 & H19 locus
chr7:149,758,083-149,858,985

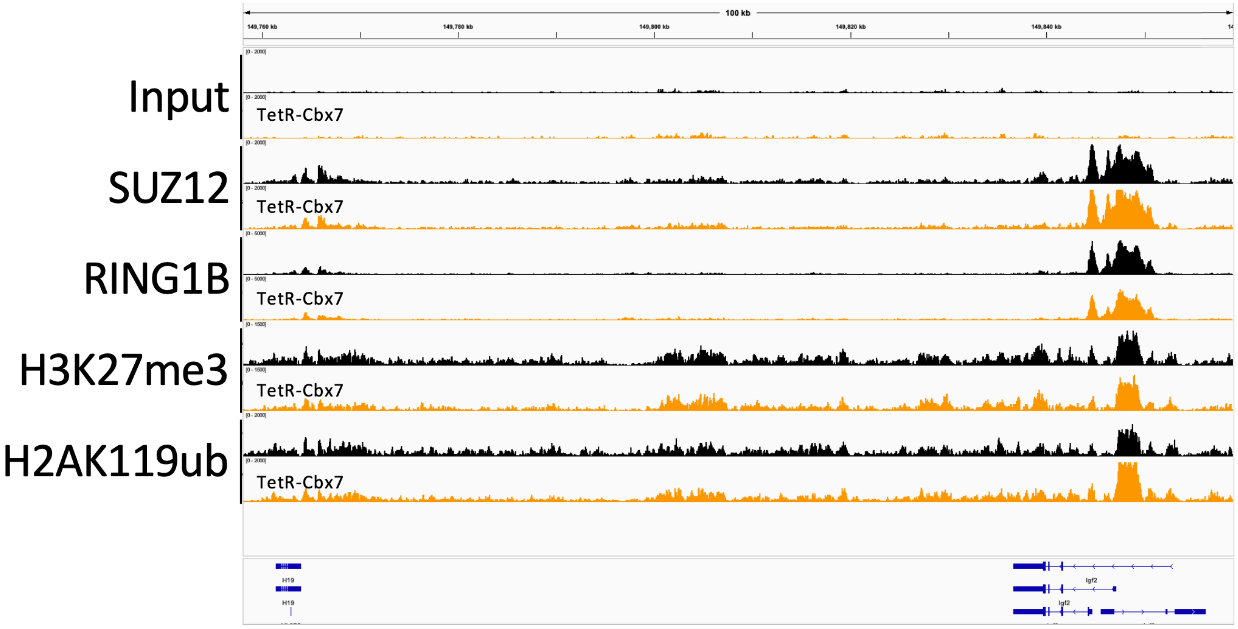

Zfp42 locus
chr8:44,361,316-44,426,512

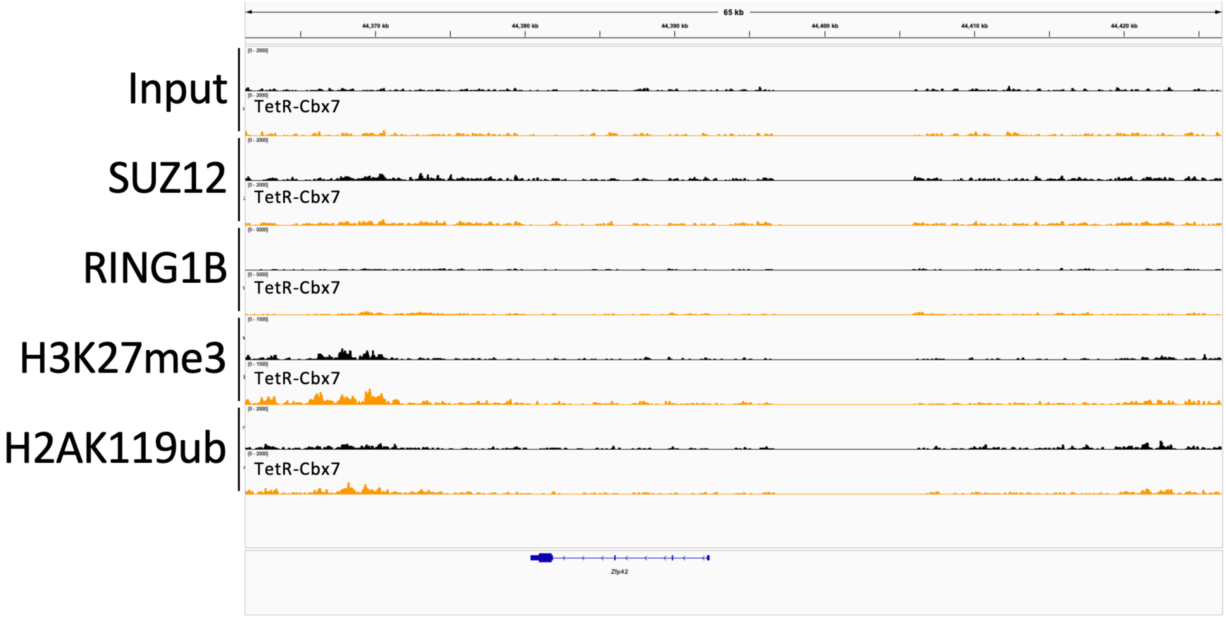

Tmed3 locus
chr9:89,592,758-89,602,918

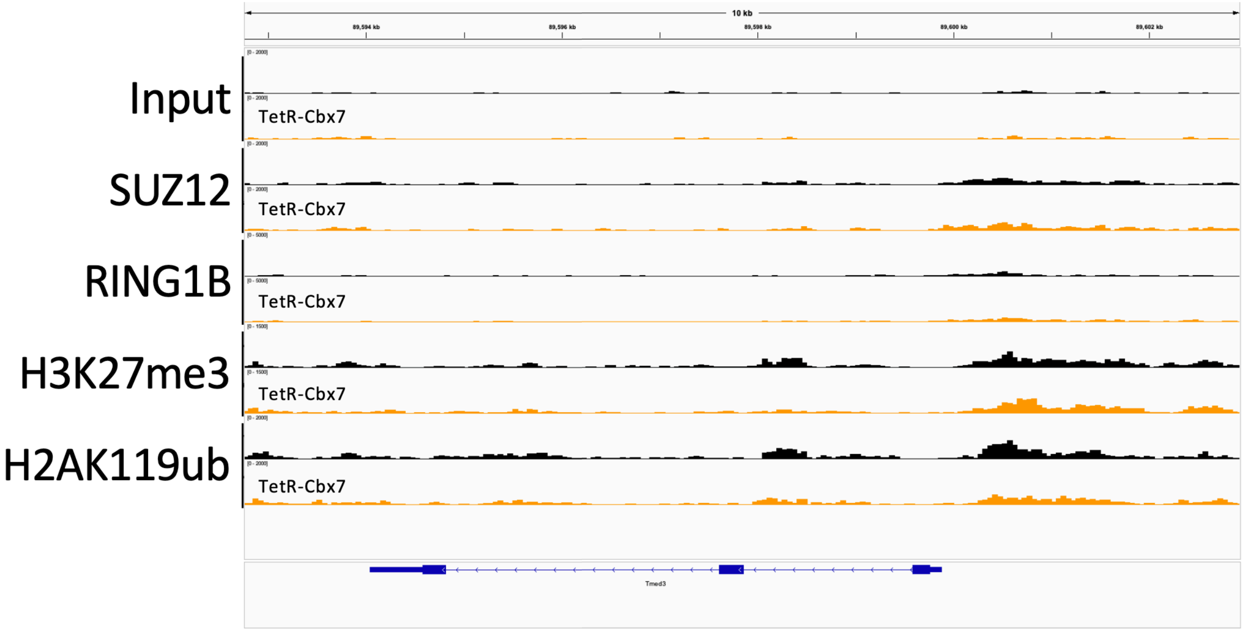

Cbx2/4/8 gene cluster
chr11:118,870,869-118,961,636

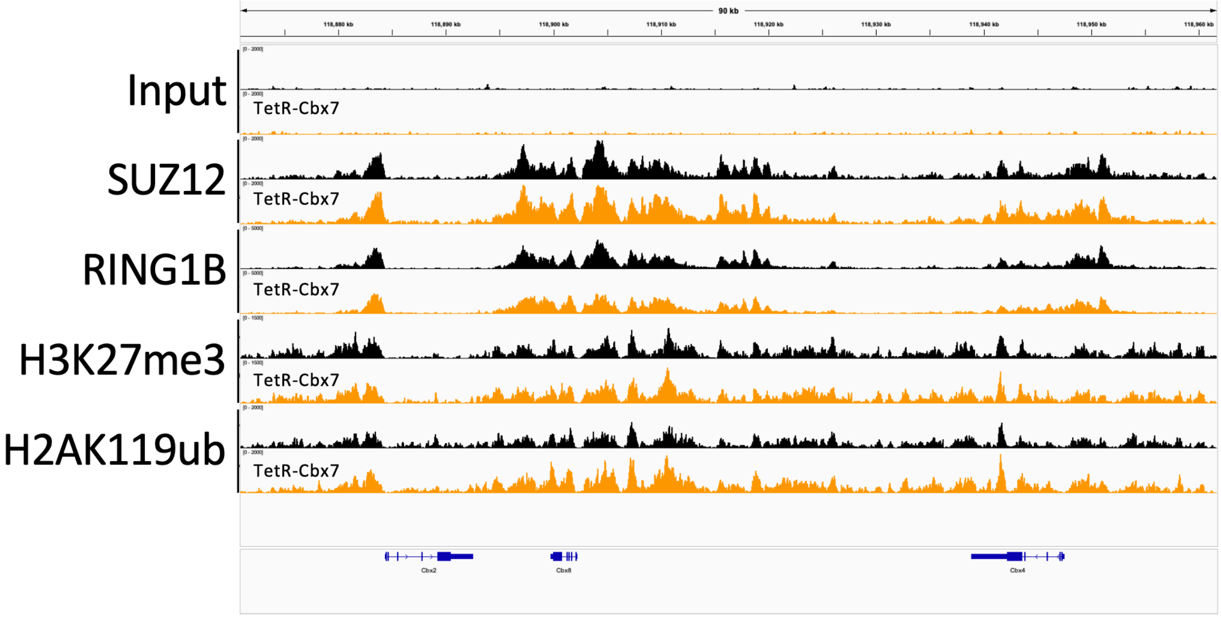

Esrrb locus
chr12:87,761,185-87,877,500

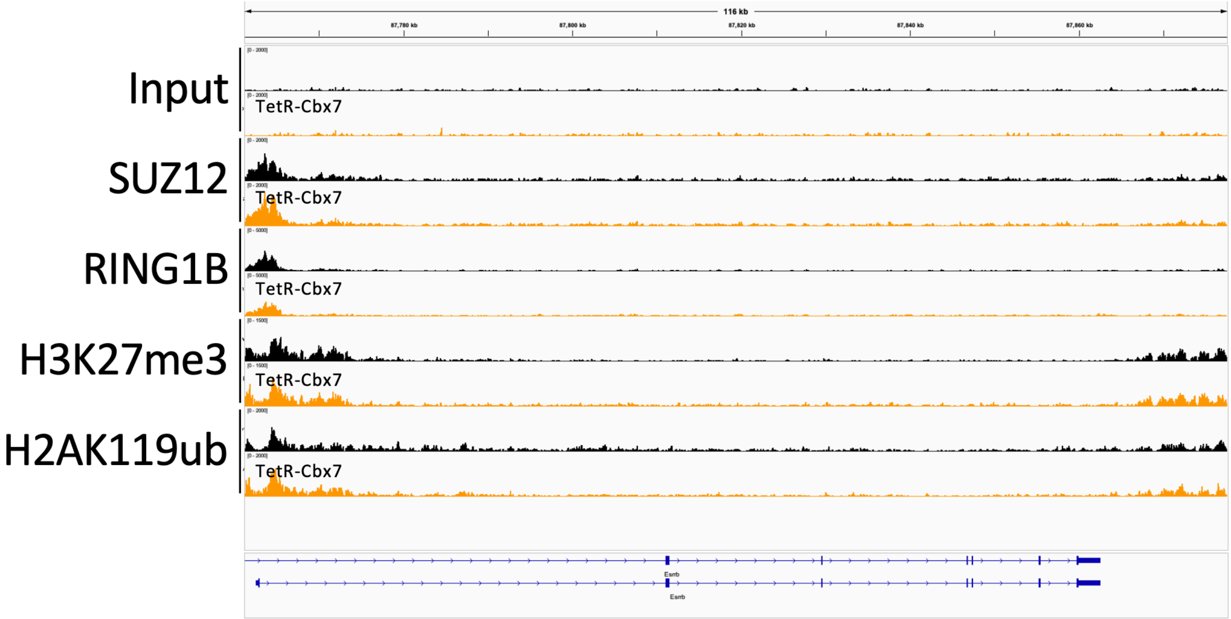

Otx2 locus
chr14:49,256,167-49,306,303

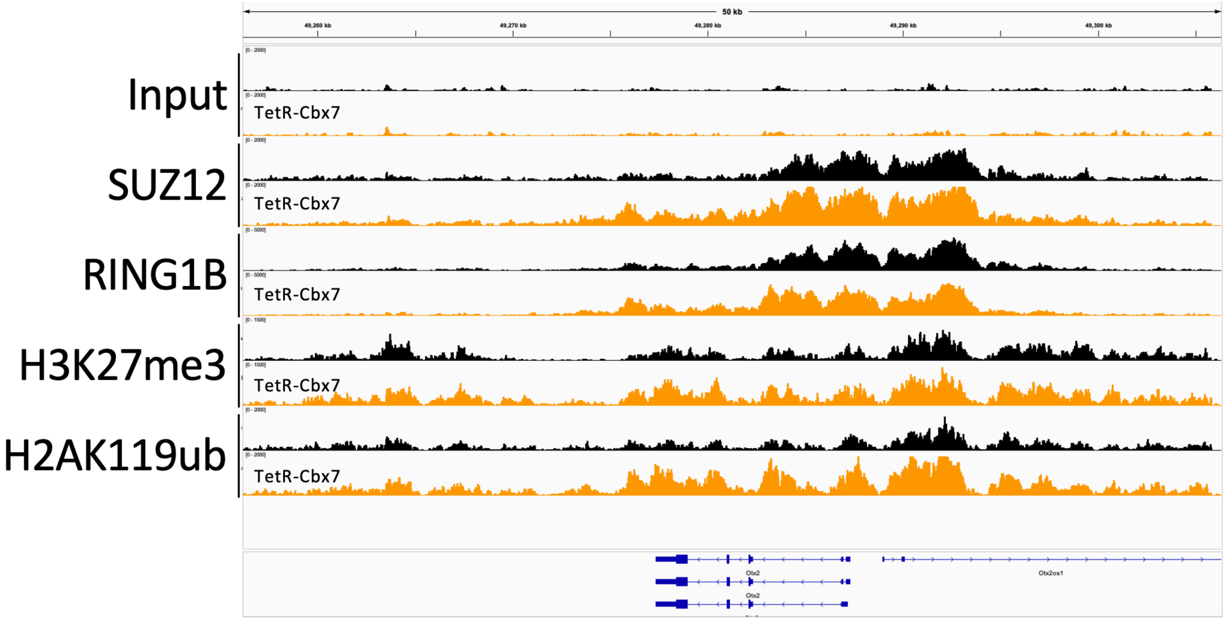

Supplement: Supplementary file 1 — Supplementary Information [file 41467_2023_43869_MOESM1_ESM.pdf]
