# Supplementary material for: Loss of cohesin regulator PDS5A reveals repressive role of Polycomb loops
Source: Nat Commun. 2023 Dec 9;14:8160. doi: 10.1038/s41467-023-43869-w (PMC10710464; doi:10.1038/s41467-023-43869-w)
Supplement: Supplementary file 3 — Reporting Summary [file 41467_2023_43869_MOESM3_ESM.pdf]

nature portfolio

Corresponding author(s): Dr Oliver Bell

Last updated by author(s): Sep 13, 2023

Reporting Summary

Nature Portfolio wishes to improve the reproducibility of the work that we publish. This form provides structure for consistency and transparency in reporting. For further information on Nature Portfolio policies, see our Editorial Policies and the Editorial Policy Checklist.

Statistics

For all statistical analyses, confirm that the following items are present in the figure legend, table legend, main text, or Methods section.

n/a	Confirmed	
☐	☒	The exact sample size (*n*) for each experimental group/condition, given as a discrete number and unit of measurement
☐	☒	A statement on whether measurements were taken from distinct samples or whether the same sample was measured repeatedly
☐	☒	The statistical test(s) used AND whether they are one- or two-sided<br>*Only common tests should be described solely by name; describe more complex techniques in the Methods section.*
☒	☐	A description of all covariates tested
☐	☒	A description of any assumptions or corrections, such as tests of normality and adjustment for multiple comparisons
☐	☒	A full description of the statistical parameters including central tendency (e.g. means) or other basic estimates (e.g. regression coefficient) AND variation (e.g. standard deviation) or associated estimates of uncertainty (e.g. confidence intervals)
☐	☒	For null hypothesis testing, the test statistic (e.g. *F*, *t*, *r*) with confidence intervals, effect sizes, degrees of freedom and *P* value noted<br>*Give P values as exact values whenever suitable.*
☒	☐	For Bayesian analysis, information on the choice of priors and Markov chain Monte Carlo settings
☒	☐	For hierarchical and complex designs, identification of the appropriate level for tests and full reporting of outcomes
☒	☐	Estimates of effect sizes (e.g. Cohen's *d*, Pearson's *r*), indicating how they were calculated

Our web collection on statistics for biologists contains articles on many of the points above.

Software and code

Policy information about availability of computer code

Data collection	Western blots were imaged on an Odyssey CLx Near-Infrared Imaging System (LICOR). FACS data was obtained on FACS LSR II Fortessa/BD Biosciences and Attune NxT flowcytometer (ThermoFIsher Scientific). NGS libraries were sequenced on HiSeq2500 and NovaSeq machines/ Illumina.
Data analysis	STAR v2.6.1c, HTSeq 0.12.3, DESeq2 v1.18.1, clusterProfiler v3.6.0, bowtie2 v2.3.4.1,, Picard MarkDuplicates v2.23.4, deepTools v3.5.0, MACS2 v2.1.1, bedtools v2.27.1, samtools v1.9, Flowjo, HiC-Pro (2.11.1), HiGlass, HiCExplorer, juicer tools (1.11.09), coolpup.py (v1.0.0), GENOVA, NGmerge, chromHMM(version 1.24)

For manuscripts utilizing custom algorithms or software that are central to the research but not yet described in published literature, software must be made available to editors and reviewers. We strongly encourage code deposition in a community repository (e.g. GitHub). See the Nature Portfolio guidelines for submitting code & software for further information.

Data

Policy information about availability of data

All manuscripts must include a data availability statement. This statement should provide the following information, where applicable:

- Accession codes, unique identifiers, or web links for publicly available datasets
- A description of any restrictions on data availability
- For clinical datasets or third party data, please ensure that the statement adheres to our policy

All NGS data reported in this study has been deposited at the Gene Expression Omnibus (GEO) database under accession number GSE194268. Source data are provided with this paper.

Research involving human participants, their data, or biological material

Policy information about studies with human participants or human data. See also policy information about sex, gender (identity/presentation), and sexual orientation and race, ethnicity and racism.

Reporting on sex and gender	does not apply
Reporting on race, ethnicity, or other socially relevant groupings	does not apply
Population characteristics	does not apply
Recruitment	does not apply
Ethics oversight	does not apply

Note that full information on the approval of the study protocol must also be provided in the manuscript.

Field-specific reporting

Please select the one below that is the best fit for your research. If you are not sure, read the appropriate sections before making your selection.

☒ Life sciences ☐ Behavioural & social sciences ☐ Ecological, evolutionary & environmental sciences

For a reference copy of the document with all sections, see nature.com/documents/nr-reporting-summary-flat.pdf

Life sciences study design

All studies must disclose on these points even when the disclosure is negative.

Sample size	No sample size calculation was performed; all experiments in this work build on established experimental schemes in the field of chromatin biology. Sample sizes are indicated throughout manuscript.
Data exclusions	No data was excluded from the analysis.
Replication	Two replicates were performed for all ChIP-seq experiments. At least three replicates were performed for all ATAC-seq experiments. Two independent replicates were performed for RNA-seq experiments. Five independent replicates were performed for Hi-C experiments. For each experiment, all attempts at replication were successful.
Randomization	Randomization is not an appropriate aspect of the experimental design presented in this study as measurements and data collection were performed using the same clonal cell lines, nucleic acid and proteins and analyzed with identical methodologies.
Blinding	Blinding was not relevant for this study as there were no prior assumptions made. All data was collected and processed uniformly regardless of sample identity. Additionally, blinding was not feasible as the investigators who performed experiments also analyzed the data.

Reporting for specific materials, systems and methods

We require information from authors about some types of materials, experimental systems and methods used in many studies. Here, indicate whether each material, system or method listed is relevant to your study. If you are not sure if a list item applies to your research, read the appropriate section before selecting a response.

Materials & experimental systems

n/a	Involved in the study
☐	☒ Antibodies
☐	☒ Eukaryotic cell lines
☒	☐ Palaeontology and archaeology
☒	☐ Animals and other organisms
☒	☐ Clinical data
☒	☐ Dual use research of concern
☒	☐ Plants

Methods

n/a	Involved in the study
☐	☒ ChIP-seq
☐	☒ Flow cytometry
☒	☐ MRI-based neuroimaging

Antibodies

Antibodies used

PDS5A (Millipore Sigma #SAB2101764), PDS5B (Bethyl Laboratories A300-537A), RING1B (Cell Signaling, D22F2), SMC3 (Bethyl Laboratories A300-060A), SUZ12 (Cell Signaling D39F6), LAMIN B1 (Abcam ab16048), H2AK119ub (Cell Signaling D27C4), H3K27me3 (Diagenode, C15410195), H3 (Abcam ab1791), a-TUBULIN (Sigma-Aldrich T9026), IRDye 800CW Goat anti-Rabbit IgG (H+L) (LICOR), IRDye 680RD Goat anti-Mouse IgG (H+L) (LICOR), PCGF1 (Abcam ab202395), RAD21 (Abcam ab992), CTCF (Millipore 070729)

Validation

All antibodies used in this study are from commercial sources. Manufacturers provided validation data. PDS5A and PDS5B were further validated by genetic loss-of-function in this manuscript.

1) Validation of PDS5A (Millipore Sigma #SAB2101764): The manufacturer states that the specificity of the antibody was tested by western blot for human, mouse, bovine, rat, rabbit, pig, horse, dog samples. Specificity of the antibody has independently been verified in this manuscript. https://www.sigmaaldrich.com/US/en/product/sigma/sab2101764#product-documentation

2) Validation of PDS5B (Bethyl Laboratories A300-537A): The manufacturer states that the specificity of the antibody was tested by western blot, immunoprecipitation, immunohistochemistry for human and mouse samples. https://www.fortislife.com/products/primary-antibodies/rabbit-anti-pds5b-antibody/BETHYL-A300-537

3) Validation of RING1B (Cell Signaling, D22F2): The manufacturer states that the specificity of the antibody was tested by western blot, immunoprecipitation, immunofluorescence, flow cytometry, ChIP and Cut&Run for human, mouse, rabbit and monkey samples. https://www.cellsignal.com/products/primary-antibodies/ring1b-d22f2-xp-rabbit-mab/5694

4) Validation of SMC3 (Bethyl Laboratories A300-060A): The manufacturer states that the specificity of the antibody was tested by western blot, immunoprecipitation, immunohistochemistry for human and mouse samples. https://www.fortislife.com/products/primary-antibodies/rabbit-anti-smc3-antibody/BETHYL-A300-060

5) Validation of SUZ12 (Cell Signaling D39F6): The manufacturer states that the specificity of the antibody was tested by western blot, immunoprecipitation, immunofluorescence, flow cytometry, ChIP and Cut&Run for human, mouse, rabbit and monkey samples. https://www.cellsignal.com/products/primary-antibodies/suz12-d39f6-xp-rabbit-mab/3737

6) Validation of LAMIN B1 (Abcam ab16048): The manufacturer states that the specificity of the antibody was tested by western blot in samples derived from Mouse, Rat and Human. https://www.abcam.com/lamin-b1-antibody-nuclear-envelope-marker-ab16048.html

7) Validation of H2AK119ub (Cell Signaling D27C4): The manufacturer states that the specificity of the antibody was tested by western blot, immunoprecipitation, immunofluorescence, flow cytometry, ChIP and Cut&Tag for human, mouse, rabbit and monkey samples. https://www.cellsignal.com/products/primary-antibodies/ubiquityl-histone-h2a-lys119-d27c4-xp-rabbit-mab/8240

8) Validation of H3K27me3 (Diagenode, C15410195): The manufacturer states that the specificity of the antibody was tested by western blotDot blotting/Peptide array, immunofluorescence, ELISA, ChIP and Cut&Tag for human, mouse, Drosophila, C. elegans, Daphnia, Arabidopsis, maize, tomato, poplar, silena latifolia, C. merolae samples. https://www.diagenode.com/en/p/h3k27me3-polyclonal-antibody-premium-50-mg-27-ml

9) Validation of H3 (Abcam ab1791): The manufacturer states that the specificity of the antibody was tested by western blot, ChIP, immunohistochemistry, immunoprecipitation and immunocytochemistry for Mouse, Rat, Human, Saccharomyces cerevisiae, Xenopus laevis, Arabidopsis thaliana, Drosophila melanogaster, Indian muntjac, Schizosaccharomyces pombe. https://www.abcam.com/products/primary-antibodies/histone-h3-antibody-nuclear-marker-and-chip-grade-ab1791.html

10) Validation of a-TUBULIN (Sigma-Aldrich T9026): The manufacturer states that the specificity of the antibody was tested by western blot, immunofluorescence staining for yeast, mouse, amphibian, human, rat, chicken, fungi, bovine. https://www.sigmaaldrich.com/US/en/product/sigma/t9026

11) Validation of IRDye 800CW Goat anti-Rabbit IgG (H+L) (LICOR)): The manufacturer states that the specificity of the antibody was tested by western blot, immunohistochemistry and microscopy. https://www.licor.com/bio/reagents/irdye-800cw-goat-anti-rabbit-igg-secondary-antibody

12) Validation of IRDye 680RD Goat anti-Mouse IgG (H+L) (LICOR): The manufacturer states that the specificity of the antibody was tested by western blot, immunohistochemistry and microscopy. https://www.licor.com/bio/reagents/irdye-680rd-goat-anti-mouse-igg-secondary-antibody

13) Validation of PCGF1 (Abcam ab202395): The manufacturer states that the specificity of the antibody was tested by western blot in samples derived from human, mouse, rat and cow. https://www.abcam.com/products/primary-antibodies/pcgf1-antibody-ab194556.html

14) Validation of RAD21 (Abcam ab992): The manufacturer states that the specificity of the antibody was tested by western blot and immunoprecipitation in samples derived from human and mouse. https://www.abcam.com/products/primary-antibodies/rad21-antibody-ab992.html

15 Validation of CTCF (Millipore 07-729): The manufacturer states that the specificity of the antibody was tested by western blot and ChIP in samples derived from human, rat, canine, mouse and primate. https://www.emdmillipore.com/US/en/product/Anti-CTCF-Antibody,MM_NF-07-729

Eukaryotic cell lines

Policy information about cell lines and Sex and Gender in Research

Cell line source(s)	All cell lines used directly in this study or for generating mutants were diploid mESCs derived from originally haploid HMSc2 termed AN3-12 (Elling et al., 2017). TetR-CBX7 reporter mESCs with 7x TetO DNA binding sites flanked by GFP and BFP reporter genes were previously described (Moussa et al., 2019). Pds5a gene-trap (GT) KO and its corresponding wild type mESCs with genetrap insertion in the non-disruptive orientation were aquired from the Haplobank repository (Cell IDs: 10388IH and 10388MH) (Elling et al., 2017).
Authentication	Genotype of cell lines was tested at the level of DNA sequence and protein.
Mycoplasma contamination	All cell lines are free of Mycoplasma contamination. Cells were regularly tested for mycoplasma.
Commonly misidentified lines (See ICLAC register)	Not used in this study.

Plants

Seed stocks	Does not apply
Novel plant genotypes	Does not apply
Authentication	Does not apply

ChIP-seq

Data deposition

☒ Confirm that both raw and final processed data have been deposited in a public database such as GEO.

☒ Confirm that you have deposited or provided access to graph files (e.g. BED files) for the called peaks.

Data access links *May remain private before publication.*	https://www.ncbi.nlm.nih.gov/geo/query/acc.cgi?acc=GSE194268
Files in database submission	GSM5831910 cChIPseq_GT_Pds5a_KO_H2AK119ub_rep1<br>GSM5831911 cChIPseq_GT_Pds5a_KO_H2AK119ub_rep2<br>GSM5831912 cChIPseq_GT_Pds5a_KO_H3K27me3_rep1<br>GSM5831913 cChIPseq_GT_Pds5a_KO_H3K27me3_rep2<br>GSM5831914 cChIPseq_GT_Pds5a_KO_Input_1<br>GSM5831915 cChIPseq_GT_Pds5a_KO_Input_2<br>GSM5831916 cChIPseq_GT_Pds5a_KO_PDS5A_rep1<br>GSM5831917 cChIPseq_GT_Pds5a_KO_PDS5A_rep2<br>GSM5831918 cChIPseq_GT_Pds5a_KO_Rad21_rep1<br>GSM5831919 cChIPseq_GT_Pds5a_KO_Rad21_rep2<br>GSM5831920 cChIPseq_GT_Pds5a_KO_Ring1B_rep1<br>GSM5831921 cChIPseq_GT_Pds5a_KO_Ring1B_rep2<br>GSM5831922 cChIPseq_GT_Pds5a_KO_Suz12_rep1<br>GSM5831923 cChIPseq_GT_Pds5a_KO_Suz12_rep2<br>GSM5831924 cChIPseq_GT_wt_Cbx7_rep1<br>GSM5831925 cChIPseq_GT_wt_Cbx7_rep2<br>GSM5831926 cChIPseq_GT_wt_CTCF_rep1<br>GSM5831927 cChIPseq_GT_wt_CTCF_rep2<br>GSM5831928 cChIPseq_GT_wt_H2AK119ub_rep1<br>GSM5831929 cChIPseq_GT_wt_H2AK119ub_rep2<br>GSM5831930 cChIPseq_GT_wt_H3K27me3_rep1<br>GSM5831931 cChIPseq_GT_wt_H3K27me3_rep2<br>GSM5831932 cChIPseq_GT_wt_Input_1<br>GSM5831933 cChIPseq_GT_wt_Input_2

GSM5831934 cChIPseq_GT_wt_Pcgf1_rep1
GSM5831935 cChIPseq_GT_wt_Pcgf1_rep2
GSM5831936 cChIPseq_GT_wt_PDS5A_rep1
GSM5831937 cChIPseq_GT_wt_PDS5A_rep2
GSM5831938 cChIPseq_GT_wt_Rad21_rep1
GSM5831939 cChIPseq_GT_wt_Rad21_rep2
GSM5831940 cChIPseq_GT_wt_Ring1B_rep1
GSM5831941 cChIPseq_GT_wt_Ring1B_rep2
GSM5831942 cChIPseq_GT_wt_Suz12_rep1
GSM5831943 cChIPseq_GT_wt_Suz12_rep2

Genome browser session
(e.g. UCSC)

he xxx.bw files can be uploaded (all at once or selected) to the UCSC genome browser by pasting all (mm10) URLs into "Paste URLs or data" in "add custom tracks".

Methodology

Replicates

ChIP-seq experiments were performed with two independent replicates.

Sequencing depth

Input_wt 30,180,572
Input_Pds5a_KO 26,672,594
H2Aub_wt_rep1 24,571,504
H2Aub_wt_rep2 27,577,968
H2Aub_Pds5a_KO_rep1 27,462,904
H2Aub_Pds5a_KO_rep2 32,058,518
K27me3_wt_rep1 47,795,516
K27me3_wt_rep2 28,411,706
K27me3_Pds5a_KO_rep1 49,772,922
K27me3_Pds5a_KO_rep2 39,035,030
Rnf2_wt_rep1 54,153,586
Rnf2_wt_rep2 49,681,830
Rnf2_Pds5a_KO_rep1 38,368,316
Rnf2_Pds5a_KO_rep2 42,130,506
Cbx7_wt_rep1 28,762,882
Cbx7_wt_rep2 27,874,380
Input_wt 53,377,272
Input_Pds5a_KO 48,292,340
Pcgf1_wt_rep1 27,490,912
Pcgf1_wt_rep2 17,250,462
CTCF_wt_rep1 18,644,566
CTCF_wt_rep2 30,558,054
Rad21_wt_rep1 28,215,532
Rad21_wt_rep2 26,980,128
Rad21_Pds5a_KO_rep1 31,529,766
Rad21_Pds5a_KO_rep2 30,020,762
Suz12_wt_rep1 20,913,958
Suz12_wt_rep2 22,088,794
Suz12_Pds5a_KO_rep1 25,144,526
Suz12_Pds5a_KO_rep2 26,569,506

Antibodies

H3K27me3 (Diagenode, C15410195), RING1B (Cell Signaling, D22F2), PDS5a (Millipore Sigma #SAB2101764), SUZ12 (Cell Signaling D39F6), H2AK119ub (Cell Signaling D27C4), PCGF1 (Abcam ab202395), RAD21 (Abcam ab992), CTCF (Millipore 070729).

Peak calling parameters

--broad --broad-cutoff 0.01 -q 0.01 -m 10 30

Data quality

We observed good library mapping rates and agreement between replicates. Spearman correlation between independent replicates is >0.8.

Software

bowtie2, SAMtools, Picard, deepTools, MACS2

Flow Cytometry

Plots

Confirm that:

☒ The axis labels state the marker and fluorochrome used (e.g. CD4-FITC).

☒ The axis scales are clearly visible. Include numbers along axes only for bottom left plot of group (a 'group' is an analysis of identical markers).

☐ All plots are contour plots with outliers or pseudocolor plots.

☐ A numerical value for number of cells or percentage (with statistics) is provided.

Methodology

Sample preparation	ES cells are trypsinized and washed prior to preparing single cell suspension for flow analysis either in tubes or 96 well format.
Instrument	FACS LSR II Fortessa/BD biosciences; Attune NxT ThermoFisher Scientific
Software	BD FACSDiva 8 for acquiring data. Flowjo software for visualization.
Cell population abundance	small fraction GFP-positive TetO reporter mESCs (<1%) were enriched by three rounds of GFP sorting. The enriched population was used for identification of dependencies required for CBX7-mediated reporter gene silencing using deep sequencing.
Gating strategy	mESCs were gated for live cells (Area FSC-Area SSC), doublets (Area FSC-Height FSC), GFP and mCherry.

☐ Tick this box to confirm that a figure exemplifying the gating strategy is provided in the Supplementary Information.
